# Defects in the cytoplasmic assembly of axonemal dynein arms cause morphological abnormalities and dysmotility in sperm cells leading to male infertility

**DOI:** 10.1371/journal.pgen.1009306

**Published:** 2021-02-26

**Authors:** Isabella Aprea, Johanna Raidt, Inga Marlena Höben, Niki Tomas Loges, Tabea Nöthe-Menchen, Petra Pennekamp, Heike Olbrich, Thomas Kaiser, Luisa Biebach, Frank Tüttelmann, Judit Horvath, Maria Schubert, Claudia Krallmann, Sabine Kliesch, Heymut Omran

**Affiliations:** 1 Department of General Pediatrics, University Hospital Muenster, Muenster, Germany; 2 Institute of Reproductive Genetics, University of Muenster, Muenster, Germany; 3 Institute of Human Genetics, University Hospital Muenster, Muenster, Germany; 4 Department of Clinical and Surgical Andrology, Centre of Reproductive Medicine and Andrology, University Hospital Muenster, Muenster, Germany; Washington University School of Medicine, UNITED STATES

## Abstract

Axonemal protein complexes, such as outer (ODA) and inner (IDA) dynein arms, are responsible for the generation and regulation of flagellar and ciliary beating. Studies in various ciliated model organisms have shown that axonemal dynein arms are first assembled in the cell cytoplasm and then delivered into axonemes during ciliogenesis. In humans, mutations in genes encoding for factors involved in this process cause structural and functional defects of motile cilia in various organs such as the airways and result in the hereditary disorder primary ciliary dyskinesia (PCD). Despite extensive knowledge about the cytoplasmic assembly of axonemal dynein arms in respiratory cilia, this process is still poorly understood in sperm flagella. To better define its clinical relevance on sperm structure and function, and thus male fertility, further investigations are required. Here we report the fertility status in different axonemal dynein preassembly mutant males (*DNAAF2/ KTU*, *DNAAF4/ DYX1C1*, *DNAAF6/ PIH1D3*, *DNAAF7/ZMYND10*, *CFAP300/C11orf70* and *LRRC6*). Besides andrological examinations, we functionally and structurally analyzed sperm flagella of affected individuals by high-speed video- and transmission electron microscopy as well as systematically compared the composition of dynein arms in sperm flagella and respiratory cilia by immunofluorescence microscopy. Furthermore, we analyzed the flagellar length in dynein preassembly mutant sperm. We found that the process of axonemal dynein preassembly is also critical in sperm, by identifying defects of ODAs and IDAs in dysmotile sperm of these individuals. Interestingly, these mutant sperm consistently show a complete loss of ODAs, while some respiratory cilia from the same individual can retain ODAs in the proximal ciliary compartment. This agrees with reports of solely one distinct ODA type in sperm, compared to two different ODA types in proximal and distal respiratory ciliary axonemes. Consistent with observations in model organisms, we also determined a significant reduction of sperm flagellar length in these individuals. These findings are relevant to subsequent studies on the function and composition of sperm flagella in PCD patients and non-syndromic infertile males. Our study contributes to a better understanding of the fertility status in PCD-affected males and should help guide genetic and andrological counselling for affected males and their families.

## Author summary

Impaired male fertility is a major issue and affects several men worldwide. Patients may present with reduced number or complete absence of sperm in the ejaculate, as well as functional and/or morphological sperm defects compromising sperm motility. Despite several diagnostic efforts, the underlying causes of these defects often remain unknown („idiopathic“). The beating of sperm flagella as well as motile cilia, such as those of the respiratory tract, is driven by dynein-based motor protein complexes, namely outer and inner dynein arms. In motile cilia these protein complexes are known to be first assembled in the cytoplasm and then delivered into the cilium. In sperm, this process is still poorly understood. Here we analyze sperm cells of male individuals with mutations in distinct genes encoding factors involved in the preassembly of these motor protein complexes. Consistent with defects in their respiratory ciliated cells, these individuals also demonstrate defects in sperm flagella that cause male infertility due to immotile sperm, with a reduction of flagellar length. Our results strengthen the assumption that the preassembly process of outer and inner dynein arms is clinically relevant also in sperm and provide knowledge that should guide genetic and andrological counselling for a subgroup of men with idiopathic infertility.

## Introduction

According to the World Health Organization (WHO), infertility is defined as the inability to conceive a child naturally after at least one year of frequent, unprotected sexual intercourse. Around 10–15% of couples worldwide suffer from infertility. Diagnostic causes are equally attributed to both the female and male partner [1–4]. Male infertility represents a highly heterogeneous pathological condition that affects approximately 7% of the male population [5] comprising various causes.

At least 2000 genes are involved in spermatogenesis and complex sperm and histological testicular phenotypes are observed in infertile men [6]. Among these quantitative and qualitative spermatogenic defects, other disturbances contribute to male infertility, such as ductal obstruction or dysfunction, and also hypothalamic-pituitary axis dysfunction [7]. Therefore, the proper diagnosis of male infertility (especially genetic analysis), still represents a challenge in most cases. Despite numerous investigative efforts, the cause and pathomechanism underlying male infertility remain unsolved in approximately 40% of cases [3]. One factor that contributes to idiopathic cases of male infertility is asthenozoospermia (reduced sperm motility) due to defective sperm flagellar function. Combinations with abnormal sperm morphology (asthenoteratozoospermia) and also reduced numbers of sperm in the ejaculate (oligoasthenoteratozoospermia) are frequently observed [3].

**Fig 1:**
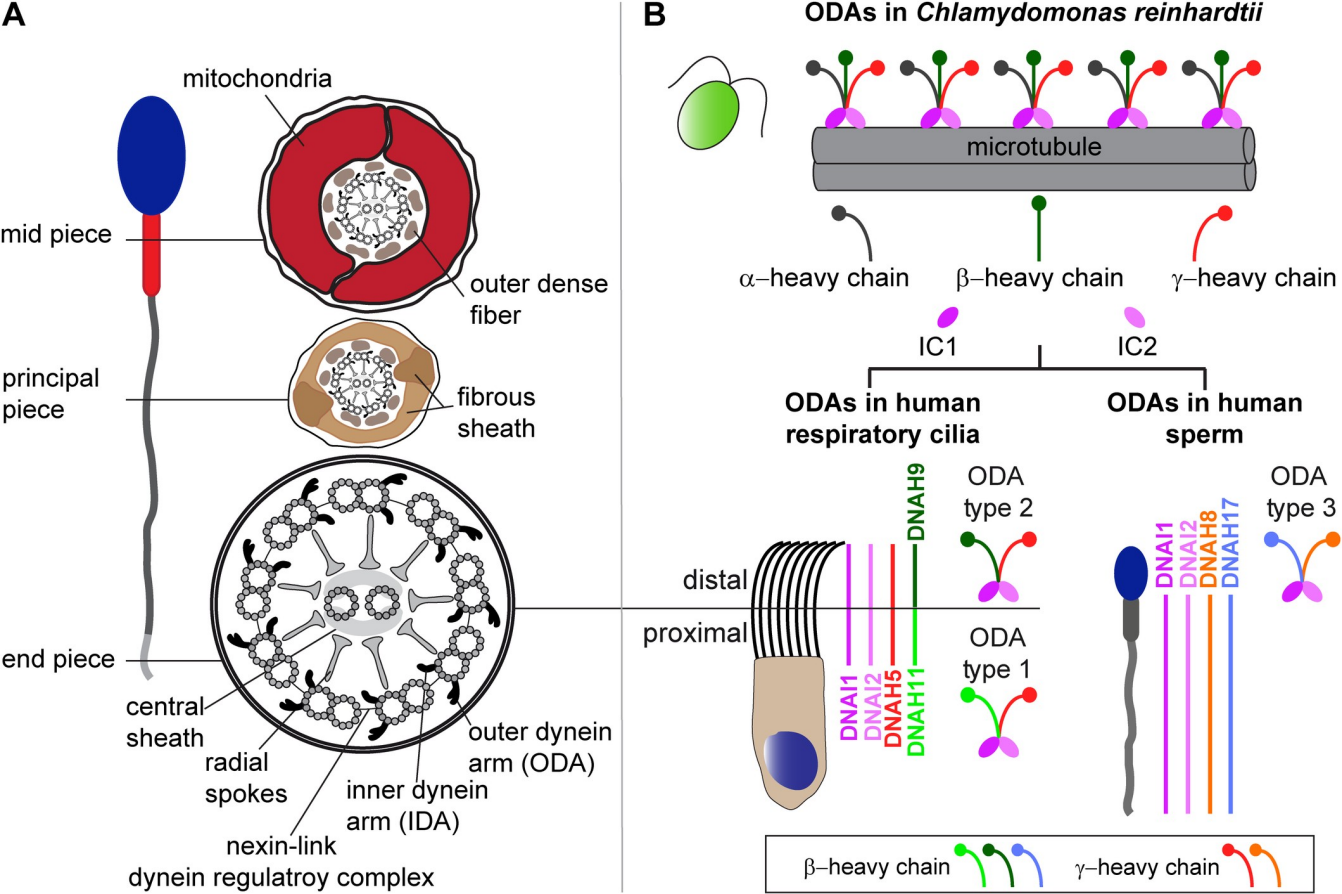
Structure of sperm flagella and respiratory cilia and ODA composition in both cell types. **(A)** Cross section schemes of a sperm flagellum and multiciliated respiratory epithelial cell. Both cell types carry a microtubule-based 9+2 axoneme and multiprotein complexes serving as functional modules. Nine outer microtubule doublets surround the central pair and are connected by the nexin-link dynein regulatory complex. Radial spokes form the connecting bridge to the central pair and its associated protein complexes, the central sheath. Each outer doublet carries outer (ODA) and inner (IDA) dynein arms, important for flagellar and ciliary beat generation and regulation. Sperm flagella carry accessory structures that define specific flagellar regions. The mid piece contains a mitochondrial sheath and outer dense fibers surrounding the axoneme. In the sperm flagellar principal piece, the mitochondrial sheath is substituted by a fibrous sheath. In the flagellar end piece, the axoneme is surrounded solely by the plasma membrane. (**B**) ODAs of the green algae *Chlamydomonas reinhardtii* present a three-headed structure with an α-, β-, and γ-heavy chain (HC). Mammals present different ODA types, all characterized by a two-headed structure. Respiratory cilia carry ODA type 1 in the proximal and ODA type 2 in the distal ciliary compartment. In comparison to *Chlamydomonas*, both mammalian ODA types carry the γ-HC ortholog DNAH5, while the β-HC orthologs DNAH11 and DNAH9 are specific to type 1 and 2, respectively. ODA type 3 is specific to sperm flagella and carries DNAH8 as γ-HC and DNAH17 as β-HC ortholog. DNAI1 and DNAI2 are orthologs of *Chlamydomonas* intermediate chains (IC1 and 2) and are present in all three ODA types [17,18].

Sperm flagella are part of the group of eukaryotic motile cilia that also include multiple motile cilia of the respiratory tract. Motile cilia perform several functions, including mucociliary clearance of the airway, the movement of cerebrospinal fluid along the brain ventricular system, supporting the transport of the oocyte to the uterus, and moving the male germ cell along the female reproductive tract [8]. Motile cilia and flagella are hair-like structures that contain 9+2 microtubule-based axonemes and several functional modules, whose coordinated activity assures the proper function of these organelles (Fig 1A) [8]. This basic 9+2 structure is remarkably conserved throughout evolution [9].

Multi-subunit motor protein complexes such as axonemal outer (ODA) and inner (IDA) dynein arms are essential for the beat generation and regulation of motile flagellar and ciliary beating, respectively. Dynein arms are ATPase-based protein-complexes (“AAA+”: ATPases associated with various cellular activities) that convert chemical energy released upon ATP binding and hydrolysis into mechanical force, enabling the sliding between outer microtubule doublets [10–15]. Composition of ODAs and IDAs is well investigated in *Chlamydomonas reinhardtii*, a unicellular green algae swimming with two flagella. The ODA in *Chlamydomonas* consists of 16 subunits. These comprise the dynein heavy chains (HC) α, β and γ, forming a globular head structure, two intermediate chains (IC) IC1 and IC2, and eleven light chains (LCs 1, 2, 3, 4, 5, 6, 7a, 7b, 8, 9, and 10) [15,16].

Our group has shown that in contrast to the singular three-headed ODA type in *Chlamydomonas*, two distinct double-headed ODA types with distinct spatial localization exist (Fig 1B) in mammals—ODA type 1 and ODA type 2. ODA type 1 comprises DNAH5 (ortholog of the γ-HC in *Chlamydomonas*) and DNAH11 (ortholog of the β-HC in *Chlamydomonas*), whereas ODA type 2 comprises DNAH5 and DNAH9 (also an ortholog of the β-HC in *Chlamydomonas*), (Fig 1B). In human respiratory cilia, ODA type 2 is localized in the distal part, whereas ODA type 1 is located in the proximal part of the ciliary axoneme [17] (Fig 1B). In contrast, a recent study demonstrated that sperm flagella contain a third ODA type that localizes along the entire sperm flagellar length [18], (Fig 1B). This ODA type 3 carries sperm-specific dynein HCs, namely DNAH8 (ortholog of the γ-HC in *Chlamydomonas*) and DNAH17 (ortholog of the β-HC in *Chlamydomonas*), that are respectively paralogs of DNAH5 and DNAH9/ DNAH11 [18], (Fig 1B).

The components of axonemal dynein arms are known to be first pre-assembled to a multi-protein complex in the cytoplasm of ciliated/ flagellated cells, and then delivered to the axoneme during cilio- and flagellogenesis [19,20]. The process of cytoplasmic preassembly of dynein arms is regulated by evolutionarily conserved proteins referred to as dynein axonemal assembly factors (DNAAFs) [21]. Dynein axonemal assembly factors have different aliases, depending on the model organism. The Human Genome Organization Gene Nomenclature Committee (HGNC) aims to provide a unique symbol for each and every human gene, preferably one which maintains a parallel construction in different members of a gene family and which can also be used for orthologous genes in other species, particularly in vertebrates [22]. To improve readability, we will focus on the current human gene symbols. An overview of different gene symbols for each DNAAF is given in Table 1.

**Table 1:** List of proteins involved in the cytoplasmic preassembly of axonemal dyneins in human and model organisms and their aliases. Crosses indicate in which model organism a cytoplasmic preassembly factor was analyzed and if mutations in the orthologous human gene were identified.

Axonemal dynein preassembly factor	Analyzed in model organisms and identified in Humans										References
	*Chlamydomonas reinhardtii*	*Paramecium tetraurelia*	*Trypanosoma brucei*	*Schmidtea mediterranea*	*Drosophila melanogaster*	*Danio rerio*	*Oryzias latipes*	*Xenopus laevi*	*Mus musculus*	*Homo sapiens*	
CFAP298/Kurly/C21orf59/ CILD26/ FBB18	x			x		x				x	[26–28]
CFAP300/C11orf70/ CILD38/FBB5	x	x								x	[29–31]
DNAAF1/LRRC50/ CILD13/ODA7	x		x			x			x	x	[32–40]
DNAAF2/KTU/Kintoun/ C14orf104/CILD10/ PF13	x					x	x		x	x	[23,25,32,42,52,69,75,78]
DNAAF3/C19orf51/ PF22/ CILD2	x					x				x	[32,41,42]
DNAAF4/DYX1C1/ DYX1/ PF23/CILD25	x					x			x	x	[42–45]
DNAAF5/HEATR2/ HTR/ CILD18	x									x	[46–48]
DNAAF6/PIH1D3/ CXorf41/TWISTER/ CILD36						x			x	x	[25,49–52]
DNAAF7/ZMYND10/ CILD22					x	x		x	x	x	[53–57]
LRRC6/DNAAF11/ TilB/Seahorse/ CILD19			x		x	x			x	x	[53,58–64]
SPAG1/DNAAF13/ CILD28						x				x	[65–67]
TTC12/CILD45										x	[68]
PIH1D1/DNAAF14/ MOT48/NOP1/ IDA10/ PIH1	x					x					[25,52,69]
PIH1D2/DNAAF15/ Pih1d2						x					[52]
RUVBL1/RVB1/Pontin/ Tip49/CrRuvBL1						x			x		[70–72]
RUVBL2/RVB2/Reptin/ Tip48						x					[71,73]
WDR92/ DNAAF10	x			x							[74–77]

The first DNAAF identified in humans was DNAAF2 [23]. Mutations in *DNAAF2* were identified in individuals with primary ciliary dyskinesia (PCD, MIM # 244400), a motile ciliopathy characterized by recurrent respiratory infections, laterality defects and impaired fertility [23,24]. In contrast to mutations in *DNAH5*, which cause solely structural defects of the ODAs [8], mutations in *DNAAF2* result in defects of ODAs and IDAs from the axoneme [23,25]. DNAAF2 localizes to the cytoplasm and interacts with DNAI2 and HSP70. Further studies in *Chlamydomonas PF13-*mutant strain revealed that mutations in *DNAAF2* block assembly of the ODA heavy chains while ODA intermediated chains accumulate in the cytoplasm. These findings indicate that DNAAF2 functions as a chaperone and is involved in early steps of dynein assembly [23].

Additional factors involved in the cytoplasmic preassembly process of dynein arms such as CFAP298 [26–28], CFAP300 [29–31], DNAAF1 [32–40], DNAAF3 [32,41,42], DNAAF4 [42–45], DNAAF5 [46–48], DNAAF6 [25,49–52], DNAAF7 [53–57], LRRC6 [53,58–64], SPAG1 [65–67], TTC12 [68], PIH1D1 [25,52,69], PIH1D2 [52], RUVBL1 [70–72], RUVBL2 [71,73] and WDR92 [74–77] were identified from analyses of *Chlamydomonas* mutant strains, genetic screens in *Paramecium*, *Schmidtea mediterranea*, *Drosophila*, *Xenopus*, Medaka and zebrafish, knockout studies in mice or through analyses of individuals with PCD with dynein arm defects (Table 1). In unicellular organisms, knockout or knockdown of the orthologous DNAAFs result in paralyzed flagella or reduced beating and swim velocity [35,42,62]. In vertebrates, mutations in genes encoding distinct DNAAFs cause randomization of the left-right body asymmetry and laterality defects including heart defects, hydrocephalus formation and infertility. Zebrafish/Medaka fish have additional phenotypes such as scoliosis and cystic kidneys [23,49,50,53,56,78,79]. These defects are consistent with defective or disturbed motile cilia/flagella function.

**Fig 2:**
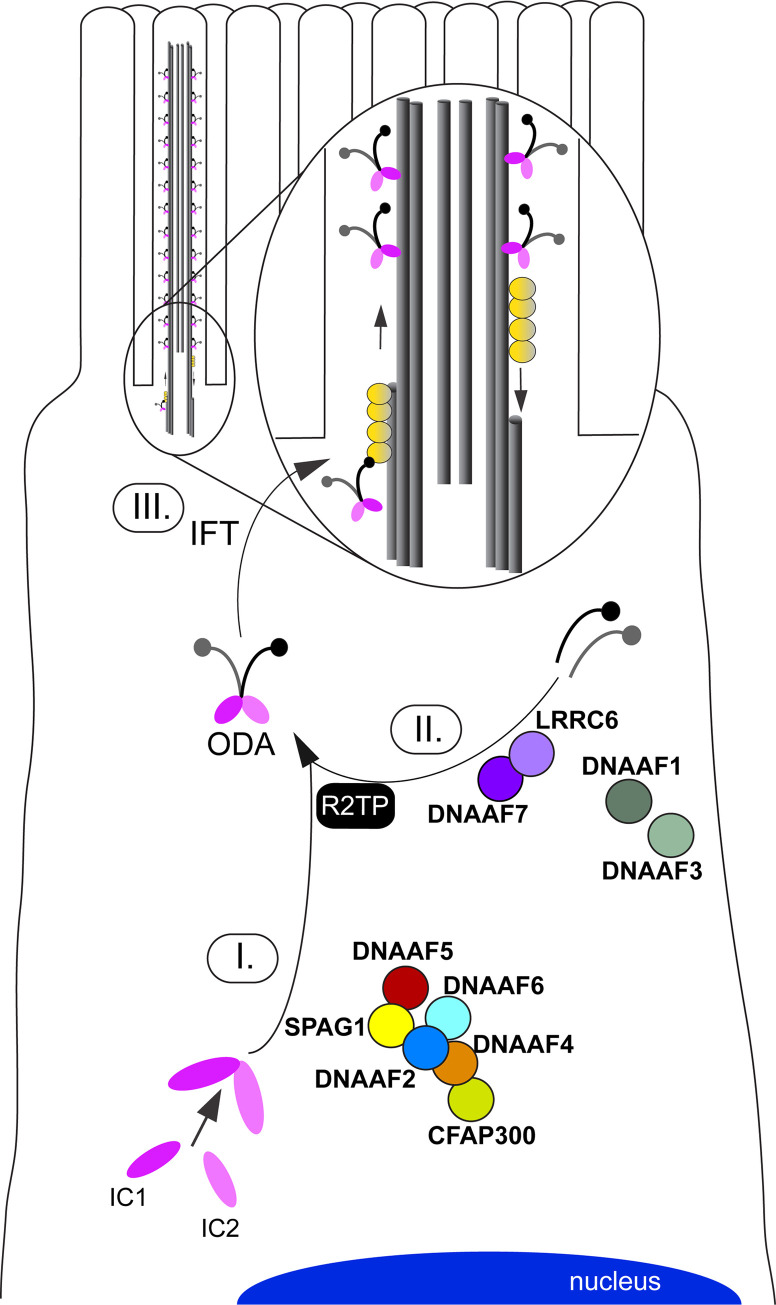
Schematic overview of the cytoplasmic dynein arm preassembly process in multiple motile cilia that is aided by distinct dynein axonemal assembly factors (DNAAFs). Motile cilia protrude from the cell surface and contain microtubule-based axonemes. During ciliogenesis, axonemal components are pre-assembled in the cytoplasm (e.g. axonemal motor protein complexes, I.+II.) and subsequently delivered to the growing axonemes (III.) by intraflagellar transport (IFT) machinery. The figure representatively shows the interactions between DNAAFs (indicated in bold) as well as distinct outer dynein arm (ODA) components, as experimentally determined in previous studies [17,29,48,50,56]. **HC**: Heavy chains; **IC**: Intermediate chains.

Functional studies in different model organisms showed that these factors act as chaperones or co-chaperones for assembly of dynein arm complexes [21]. While DNAAFs such as DNAAF2, DNAAF4 and DNAAF6 are involved in assembly and stabilization of the IC complex [23,43,50], other DNAAFs such as DNAAF7 and LRRC6 are involved in maturation of the HC subunits and transfer of the HC complex to the R2TP complex for stable association with the IC complex (Fig 2), [56]. For further information on the cytoplasmic preassembly of axonemal dyneins we refer to reference [21].

Despite extensive knowledge about the cytoplasmic dynein preassembly process in multiciliated cells of the airways, this process is still not fully understood, especially in other ciliated/flagellated cell types such as sperm cells. Therefore, we studied the fertility status of male PCD individuals carrying mutations in *DNAAF2*, *DNAAF4*, *DNAAF6*, *DNAAF7*, *CFAP300* and *LRRC6* and systematically compared the composition of ODA and IDA components in sperm flagella and respiratory cilia by high-resolution immunofluorescence and transmission electron microscopy.

## Results

### Clinical characteristics and fertility status of patient cohort

All individuals included in the study cohort showed classical respiratory PCD symptoms including chronic wet cough and chronic nasal congestion (Table 2 and S1 and S2 Figs). Four of these individuals had respiratory distress after birth. Additionally, six of the nine males display *situs inversus totalis* (S1 and S2 Figs) consistent with randomization of left/right body asymmetry caused by DNAAF defects [23,29]. Three of the families are consanguineous (for OP-3399 see pedigree in S1 Fig, [23]). Diagnostic work-up according to criteria of the ERS guidelines for PCD [80] confirmed PCD diagnosis of all analyzed individuals. This includes transmission electron- (TEM), high resolution immunofluorescence- (IF) and high-speed video microscopy (HVMA) analysis of multiciliated respiratory epithelial cells, nasal nitric oxide (nNO) production rate, and genotyping (Table 2). In seven of nine individuals, measurements of nNO production rate were available. All seven males showed a nNO production rate below the cut-off value of 77 nl/min [81]. HVMA of respiratory cilia revealed immotile cilia in eight individuals and minimal residual ciliary motility in one individual (S1–S6 Videos and [23,29,50]).

**Table 2:** Clinical and diagnostic characteristics of study cohort. HVMA, high-speed video microscopy analysis; IF, high-resolution immunofluorescence microscopy; n.a., not applicable; nNO, nasal nitric oxide productionrate; ODA, outer dynein arm; RDS, respiratory distress syndrome; resp., respiratory; SI, *situs inversus totalis*; TEM, transmission electron microscopy.

Affected individual	Gene/ mutation	IF defect (resp. cells)	TEM defect (resp. cells)	HVMA (resp. cells)	nNO (nl/min)	Chronic nasal congestion	Neonatal period	SI	Consanguinity	Chronic otitis media	Reccurent respiratory infections/ wet cough	Impaired fertility
OP-146 II3	*DNAAF2*: c.1214ˆ1215 insACGATACCTGCGTGGC, p.Gly406Argfs*89 (homozygous)	DNAI1 and DNAI2 reduced, DNALI1 absent	ODA	immotile	n.a.	yes	RDS	no	yes	yes	yes	yes
OP-234 II1	*DNAAF2*: c.23C>A, p.Ser8* (homozygous)	DNAI1 and DNAI2 reduced, DNALI1 absent	ODA	immotile	7.6	yes	RDS	yes	yes	yes	yes	n.a.
OP-6 II5	*DNAAF7***:** c.47T>G, p.Val16Gly (homozygous)	DNAI1 and DNAI2 reduced, DNALI1 absent	ODA	minimal residual movement	n.a.	yes	no problems reported	no	no	yes	yes	yes
OP-596 II2	*DNAAF7*: c.47T>G, p.Val16Gly + c.490dupC, p.Gln164Profs*19	DNAI1 and DNAI2 reduced, DNALI1 absent	ODA	immotile	33.2	yes	RDS	yes	no	yes	yes	yes
OP-3399	*DNAAF4*: c.31 C>T, p.Gln11* (homozygous)	DNAI1, DNAI2, DNALI1 absent	ODA	immotile	12.7	yes	no problems reported	yes	yes	no	yes	yes
OP-1899 II1	DNAAF6: c.355C>T, p.Gln119* (hemizygous, X-linked)	DNAI1, DNAI2, DNALI1 absent	ODA	immotile	38	yes	RDS	yes	no	yes	yes	yes
OP-3141	Deletion in *DNAAF6* (hemizygous, X-linked)	DNAI1, DNAI2, DNALI1 absent	ODA	immotile	22.5	yes	no problems reported	no	no	yes	yes	yes
OP-2334	*CFAP300*: c.361C>T, p.Arg121* (homozygous)	DNAI1, DNAI2, DNALI1 absent	ODA	immotile	29.8	yes	no problems reported	yes	no	yes	yes	yes
OP-516	*LRRC6*: c.630delG, p.Trp210Cysfs*12 + c.436G>C, p.Asp146His	DNAI1, DNAI2, DNALI1 absent	ODA	immotile	22.6	yes	no problems reported	yes	no	yes	yes	yes

Eight males from our study cohort reported fertility problems, while fertility status of OP-234 II1 remains unknown. HVMA of sperm flagella from all nine individuals displayed immotile sperm flagella (S7–S12 Videos and [23,29,50]).

Four individuals (OP-516, OP-1899 II1, OP-3141 and OP-3399) additionally received andrological examinations at the Centre of Reproductive Andrology (CeRA). Consistent with an oligoasthenoteratozoospermia, OP-516 presented a reduced sperm count (<4 million sperm/ejaculate) with immotile sperm and abnormal morphology but a high percentage of vital sperm (S1 Appendix). Moreover, OP-516 presented normal findings in the andrological examination including hormonal parameters. Semen analysis of OP-1899 II1 revealed asthenozoospermia, characterized by sperm count (>75 million sperm/ejaculate) and morphology within normal range, but impaired motility and reduced vitality of sperm cells (S1 Appendix). Hormonal parameters did not show abnormal findings. Semen analysis of OP-3399 revealed normal sperm count (>100 million sperm/ejaculate) and vitality within normal range (S1 Appendix), but impaired motility and morphology, consistent with asthenoteratozoospermia. Andrological examinations including hormonal parameters did not show abnormal results. Semen analysis of OP-3141 revealed a low sperm count (4.6 million sperm/ejaculate), impaired motility, abnormal morphology resulting in oligoasthenoteratozoospermia, and reduced vitality (S1 Appendix). Hormonal parameters of OP-3141 were within normal range, but ultrasound revealed a prominent rete testis and epididymis. Clinical and diagnostic features of all individuals are summarized in Table 2 and S1 Appendix.

### Genetic testing

**Fig 3:**
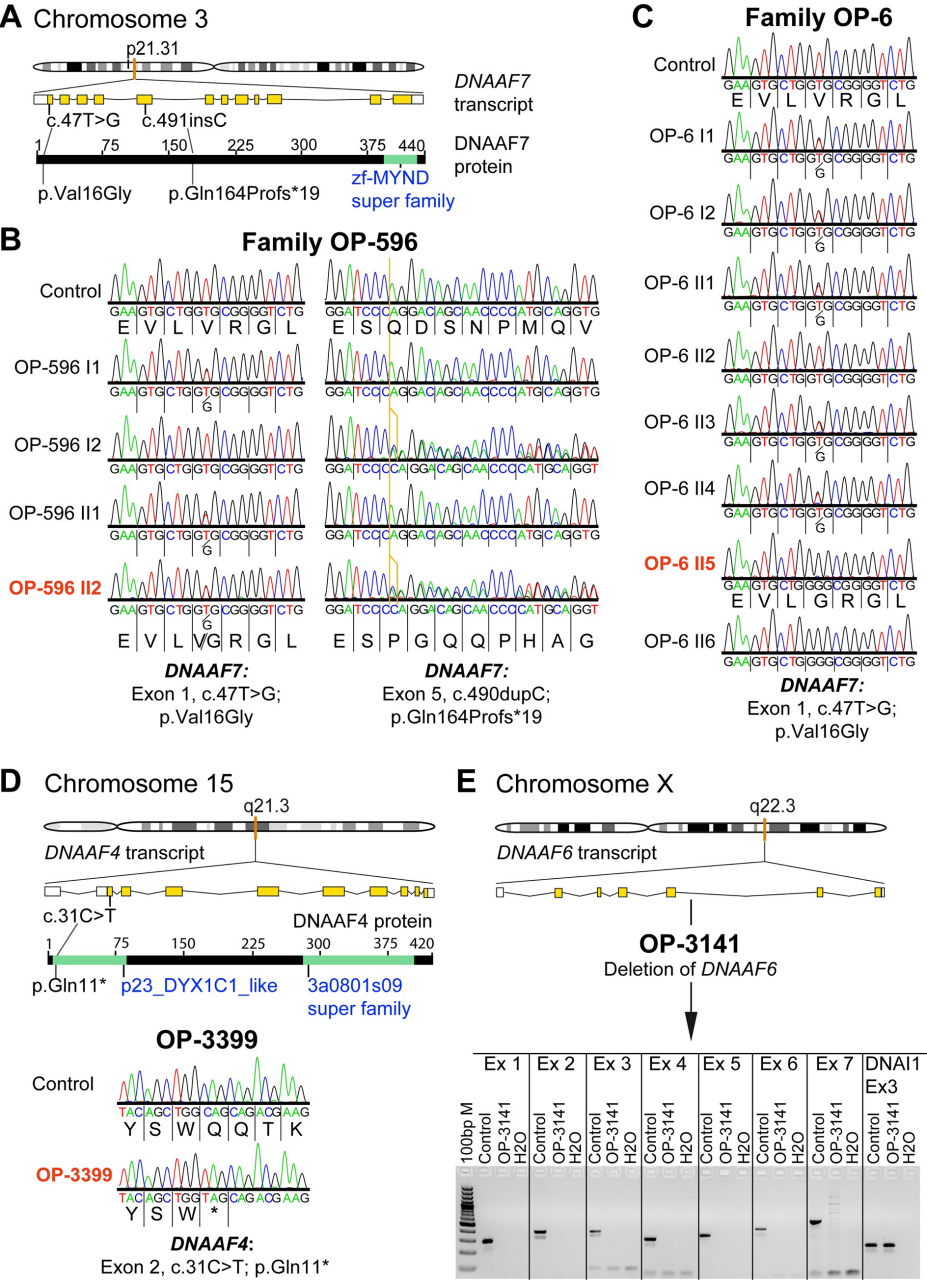
Genetic testing in families OP-6, OP-596 and individuals OP-3141 and OP-3399 identifies disease causing mutations in the genes *DNAAF7*, *DNAAF4* and *DNAAF6*, encoding axonemal dynein assembly factors. **(A)** *DNAAF7* (CCDS2825.1) is located on chromosome 3p21.31 (orange mark in the chromosome schematic) and consists of 12 exons encoding a 440 amino acid protein. A testis specific isoform comprising only 11 exons, due to selection of an alternative splicing acceptor site is annotated (CCDS77747.1). **(B)** Sanger sequencing of PCD family OP-596 confirms *DNAAF7* compound heterozygous missense and insertion mutations (Exon 1: c.47T>G; p.Val16Gly and Exon 5: c.490dupC; p.Gln164Profs*19) in affected individual OP-596 II2 (indicated in dark orange). **(C)** Sanger sequencing of PCD family OP-6 confirms a *DNAAF7* homozygous missense mutation (Exon 1: c.47T>G; p.Val16Gly) in affected individual OP-6 II5 (indicated in dark orange) and affected sibling OP-6 II6. **(D)** *DNAAF4* (CCDS10154) is located on chromosome 15q21.3 (orange mark in the chromosome schematic) and consists of one non-coding and nine coding exons, encoding a 420 amino acid protein. Sanger sequencing (bottom of the figure) for PCD affected individual OP-3399 confirms a homozygous nonsense mutation in Exon 2 (c.31C>T; p.Gln11*). **(E)** *DNAAF6* (CCDS14528) is located on the chromosome Xq22.3 (orange mark in the chromosome schematic) and consists of two non-coding and six coding exons, encoding a 214 amino acid protein. PCR amplification (lower figure in E) confirms a deletion of all *DNAAF6* exons in PCD affected individual OP-3141 compared to control. To demonstrate DNA integrity of OP-3141 we amplified Exon 3 from the *DNAI1* (ic: internal control) gene in OP-3141 and the control. Ex: exon; ins: insertion.

Genetic diagnosis of OP-146 II3, OP-234 II1, OP-1889 II1 and OP-2334 was reported previously [23,29,50], whereas the genetic diagnosis of five individuals (OP-6 II5, OP-596 II2, OP-3399, OP-3141 and OP-516) was resolved in this study. Using a diagnostic IF screening, these individuals were initially diagnosed with an ODA defect and selected for further genetic investigations. In two PCD-affected individuals, we identified disease causing variants in *DNAAF7* (CCDS2825.1; GenBank: NM_015896.4). In OP-596 II2 we identified compound heterozygous variants: a previously reported transition (c.47T>G, p.Val16Gly; rs138815960;) [53,82] and a duplication (c.490dupC), resulting in a frameshift and predicted premature stop of translation (p.Gln164Profs*19; Fig 3A and 3B).

In siblings OP-6 II5 and OP-6 II6, we identified the same missense variant [53,82] in a homozygous state (Fig 3A–3C). DNAAF7 contains a zinc finger MYND-type functional domain at the C-terminus [83]. This region is known to be crucial for interaction with LRRC6, forming a chaperone mediated complex that stabilizes dynein HCs [53,56]. It has been experimentally proven that the reported amino acid substitution (p.Val16Gly), located at the N-terminus, does not disturb the interaction with LRRC6 [53]. However, it is predicted to be probably damaging by Polymorphism Phenotyping analysis (Polyphen-2 score: 0.998) [82]. By contrast, the truncating protein alteration (p.Gln164Profs*19) is predicted to alter the interaction with LRRC6, due to its location before the C-terminal region, containing the functional domain and interaction sites for LRRC6. This single nucleotide variation (c.47T>G) has a frequency below 0.1% in dbSNP, GnomAD, ExAc, 1000 Genomes Project, Trans-Omics for Precision Medicine (TOPMed) program as well as the Exome Sequencing Project (GoESP), sponsored by the National Institutes of Health (NIH) and National Heart, Lung and Blood Institute (NHLBI) [84–87]. By contrast, the frameshift stop mutation (c.490dupC, p.Gln164Profs*19) was absent in all of these databases. In OP-3399 we identified disease causing mutations in *DNAAF4* (CCDS10154; GenBank: NM_130810.4): a homozygous transition (c.31C>T, p.Gln11*; rs1302509857) resulting in a stop codon and a predicted premature termination of translation (Fig 3D). As predicted in *Chlamydomonas*, PF23/DNAAF4 contains a CS-domain and several TPR (tetratricopeptide repeat) motifs, known to be crucial for protein-protein interaction [44]. DNAAF2 is a reported interaction partner of DNAAF4 [43]. The identified truncating protein alteration (p.Gln11*) is located at the beginning of the N-terminus and therefore predicted to abrogate these interactions. The single nucleotide variation c.31C>T has a frequency below 0.1% in dbSNP, GnomAD, 1000 Genomes Project and the Trans-Omics for Precision Medicine (TOPMed) program sponsored by the National Institutes of Health (NIH) [84,85,87]. The pedigree of OP-3141 (S1 Fig) was highly suggestive for an X-linked inheritance pattern, e.g. the dynein preassembly gene *DNAAF6* (CCDS14528; GenBank: NM_173494.2). Therefore, we first amplified all seven exons of *DNAAF6* by standard PCR for subsequent Sanger sequencing in OP-3141. Surprisingly, none of the exons could be amplified, indicating the complete deletion of *DNAAF6* in this individual (Fig 3E and S1 Table). DNAAF6, such as the dynein preassembly factor DNAAF2, contains the PIH1 domain, known to be involved in the cytoplasmic preassembly of axonemal dyneins [49,50,69]. The deletion of this crucial domain accounts for defective dynein preassembly. For OP-516, compound heterozygous mutations in *LRRC6* (CCDS6365; GenBank: NM_012472.6) were identified: a previously reported missense variant (c.436G>C, p.Asp146His; Polyphen-2 score: 1.000, probably damaging; rs200321595) and a reported single base deletion (c.630delG, p.Trp210Cysfs*12; rs760123202) leading to a disruption of the translational reading frame and a premature translational stop after twelve codons (S2 Fig) [53]. LRRC6 contains six N-terminal LRR repeats, an LRRcap domain and a CS-like domain near the C-terminus [83]. The reported amino-acid substitution (p.Asp146His) falls in the LRR cap domain, which is important for protein-protein interaction. The truncating protein variation experimentally resulted to abrogate the interaction to DNAAF7 [53]. Both genetic variations (c.436G>C and c.630delG) have a frequency below 0.1% in dbSNP, GnomAD, ExAc, 1000 Genomes Project, Trans-Omics for Precision Medicine (TOPMed) program as well as the Exome Sequencing Project (GoESP), sponsored by the National Institutes of Health (NIH) and National Heart, Lung and Blood Institute (NHLBI) [84–87]. All variants identified in this study by next generation sequencing methods were verified using Sanger sequencing (Figs 3 and S2 and S2 Table). Additionally, segregation of these variants was confirmed in families OP-6 and OP-596 (S1 Fig), whereas for OP-3141, OP-3399 and OP-516 parental DNA and/ or genetic material from siblings was not available, precluding segregation analysis.

### Preassembly mutant individuals display conserved outer and inner dynein arm defects in sperm flagella and respiratory cilia

**Fig 4:**
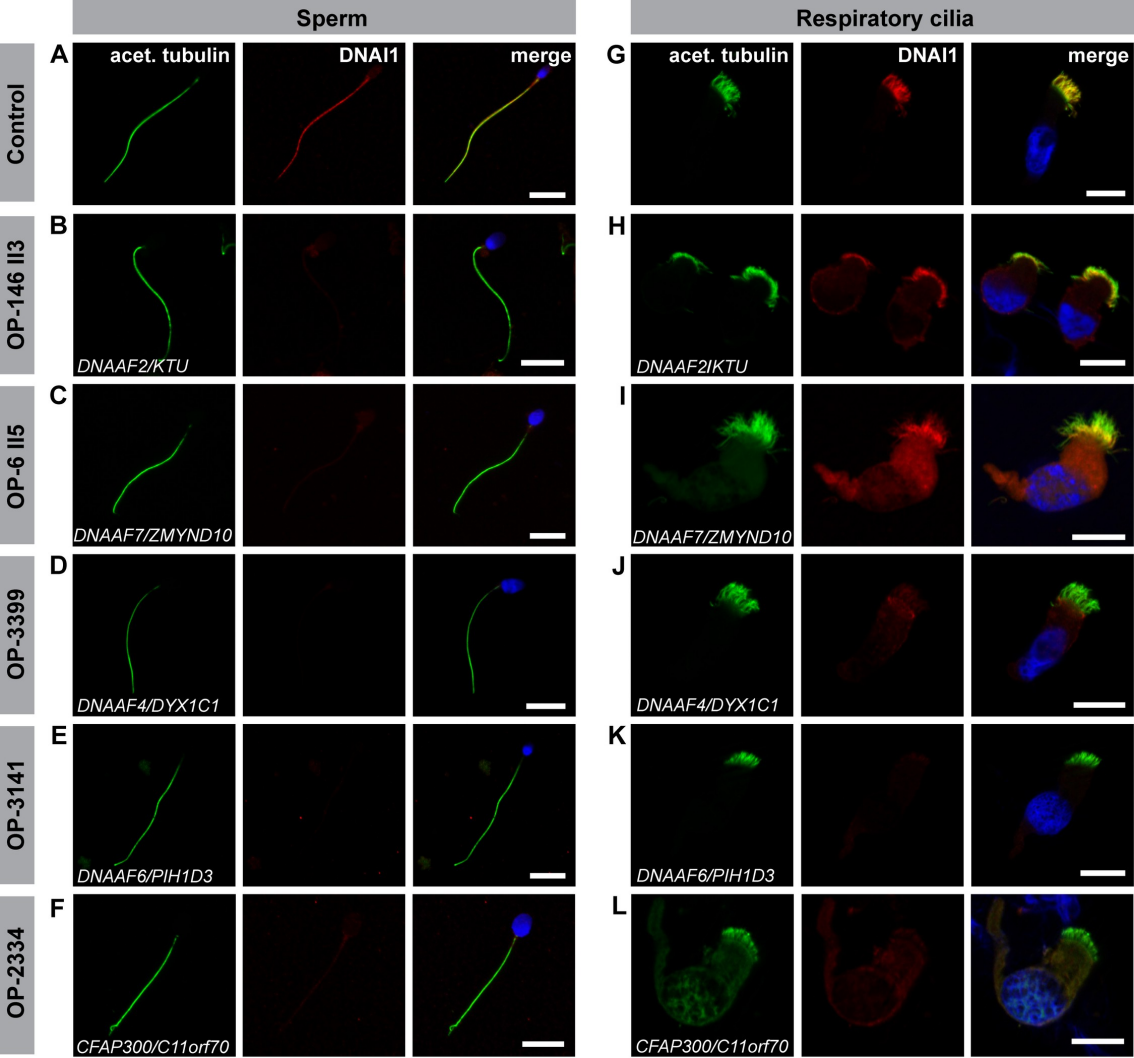
Dynein preassembly mutant sperm flagella and respiratory cilia show absence or severe reduction of the ODA intermediate chain DNAI1. Control **(A)** and mutant **(B-F)** sperm cells, as well as control **(G)** and mutant **(H-L)** respiratory cells were double-labeled with antibodies directed against acetylated α tubulin (green) and the outer dynein arm intermediate chain DNAI1 (red). Both antibodies co-localize along the flagella and cilia in cells from the healthy control (yellow, **A, G**). In all mutant sperm cells, DNAI1 is not detected along the flagellar axoneme **(B-F)**. In mutant respiratory cells of OP-146 II3 **(H)** and OP-6 II5 **(I)**, DNAI1 is retained in the proximal ciliary length, whereas in mutant cells of OP-3399 **(J)**, OP-3141 (**K**) and OP-2334 (**L**) DNAI1 displayed no immunoreactivity along the ciliary axoneme. Nuclei are stained with Hoechst33342 (blue). Scale bars represent 10 μm.

**Fig 5:**
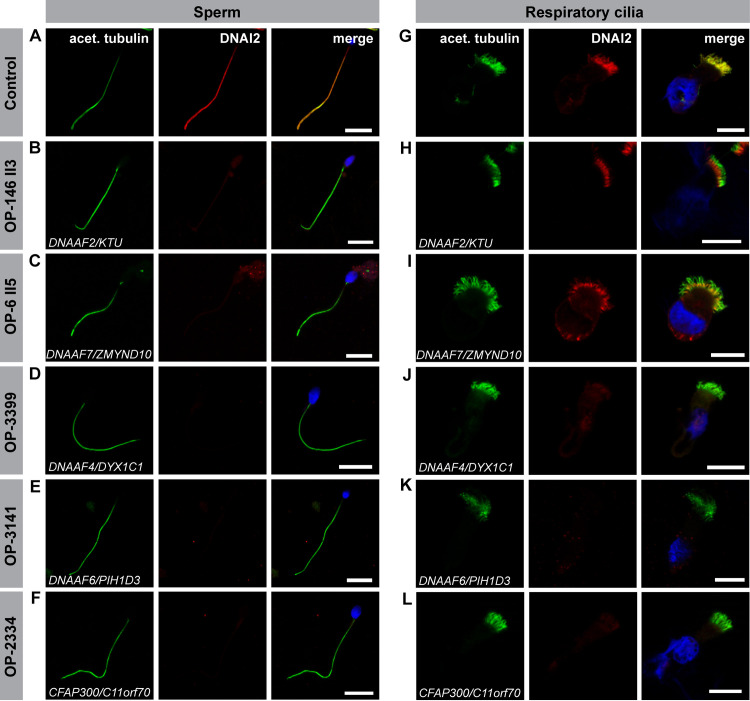
Dynein preassembly mutant sperm flagella and respiratory cilia show absence or reduction of the ODA intermediate chain DNAI2. Control **(A)** and mutant **(B-F)** sperm, as well as control **(G)** and mutant **(H-L)** respiratory cells are double-labeled with antibodies directed against acetylated α tubulin (green) and the outer dynein arm intermediate chain DNAI2 (red). Both antibodies co-localize along the flagella and cilia in cells from the unaffected control (yellow, **A, G**). In all mutant sperm cells no signal for DNAI2 is observed in flagellar axonemes **(B-F)**. In mutant respiratory cells of OP-146 II3 **(H)** and OP-6 II5 **(I)**, DNAI2 localizes to the proximal part of the ciliary axoneme, whereas in mutant cells of OP-3399 **(J)**, OP-3141 (**K**) and OP-2334 (**L**) immunoreactivity to DNAI2 is not observed in the ciliary axoneme. Nuclei are stained with Hoechst33342 (blue). Scale bars represent 10 μm.

For a comparative evaluation of ODA and IDA composition in sperm flagella and respiratory cilia in dynein preassembly mutants, we systematically performed high-resolution immunofluorescence (IF) microscopy analyses in both cell types. To investigate ODA defects, both, control and mutant sperm cells, as well as control and mutant ciliated respiratory cells were double-labeled with antibodies directed against acetylated α tubulin (flagellar and ciliary marker) and ODA intermediate chain DNAI1 and DNAI2. For *DNAAF2-*mutant (OP-146 II3 and OP-234 II1) and *DNAAF7*-mutant (OP-6 II5 and OP-596 II2) individuals we observed no immunoreactivity for the ODA marker DNAI1 and DNAI2 in sperm flagella, whereas respiratory cilia from the same individuals retained DNAI1 and DNAI2 in the proximal ciliary region (Figs 4, 5, S3 and S4 and Table 2). The *DNAAF4*-mutant (OP-3399), *DNAAF6-*mutant (OP-3141 and OP-1899 II1), *CFAP300*-mutant (OP-2334) and *LRRC6*-mutant (OP-516) individuals did not display immunoreactivity to DNAI1 and DNAI2 neither in sperm flagella nor in respiratory cilia (Figs 4, 5, S3 and S4 and Table 2).

**Fig 6:**
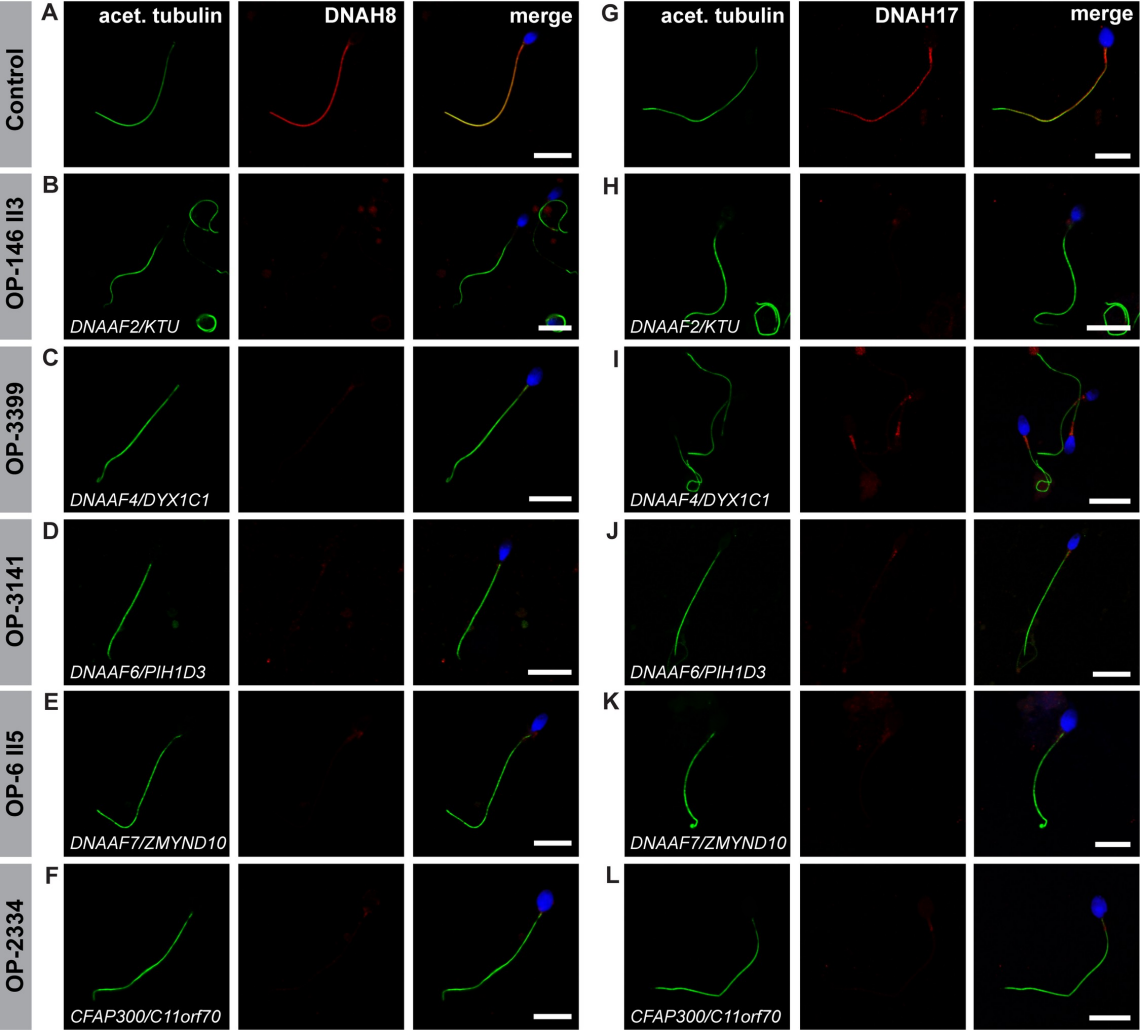
Dynein preassembly mutant sperm flagella show absence or severe reduction of the sperm specific ODA heavy chains DNAH8 and DNAH17. Control (**A** and **G**) and mutant sperm (**B-F** and **H-L**), deficient for the dynein preassembly factors DNAAF2, DNAAF4, DNAAF6, DNAAF7 and CFAP300, were double-labeled with antibodies directed against acetylated α tubulin (green), as flagellar marker, and the ODA heavy chains DNAH8 and DNAH17 (red). Both antibodies co-localize along the flagella from the unaffected control (merged image, **A, G**). In all mutant sperm flagella DNAH8 is absent from the flagellar axoneme (**B-F**). DNAH17 is either absent from the flagellar axoneme of mutant sperm (**H, K, L**) or severely reduced, displaying a residual signal in the flagellar midpiece (**I, J**), as observed in DNAAF4- and DNAAF6-deficient sperm. Nuclei are stained with Hoechst33342 (blue). Scale bars represent 10 μm.

To further characterize the ODA-defect in sperm flagella, we next analyzed the localization of the sperm specific ODA HCs, DNAH8 and DNAH17. Double-labeling with an antibody against acetylated α tubulin (flagellar marker) showed that in all mutant sperm DNAH8 is absent from the flagellar axoneme (Figs 6A–6F and S5). The localization of DNAH17 was also abnormal, displaying either absence or severe reduction (with a residual signal in the sperm flagellar midpiece) from the mutant sperm flagellar axonemes (Figs 6G–6L and S5).

The proximal localization of DNAI1 and DNAI2 in respiratory cilia of *DNAAF2*- and *DNAAF7*-mutant individuals prompted us to quantify this finding in both sperm flagella and respiratory cilia. This enabled us to investigate if in sperm cells a retention of ODA components occurs and if also in other dynein preassembly mutant individuals ODA components are retained to a certain percentage along the ciliary axoneme. We performed quantifications in at least two independent experiments and counted more than 100 cells when possible. For sperm, our analysis revealed that in over 90% of control sperm cells, DNAI1 (97%, 367 cells) and DNAI2 (95%, 347 cells) localized along the entire flagellar length. Remaining cells did not show immunoreactivity against both ODA ICs. By contrast, in all dynein preassembly mutant individuals analyzed, DNAI1 and DNAI2 was not detected in 100% of sperm cells (S6 and S7 Figs).

For nasal brush biopsies, we observed a panaxonemal DNAI1 signal in 98% (332 cells) and a panaxonemal DNAI2 signal in 92% (349 cells) of control ciliated respiratory cells (S8 and S9 Figs). Interestingly, in the *DNAAF2*-mutant individuals OP-146II3 and OP-243II1 we observed a proximal localization of DNAI1 in respectively 78% (64 counted cells) and 89% (108 counted cells) ciliated respiratory cells. The remaining amount of cells (22% for OP-146 II3 and 11% for OP-234 II1) did not display immunoreactivity against DNAI1 (S8 Fig). For OP-146 II3 and OP-234 II1, DNAI2 localized to the proximal ciliary region respectively in 74% (38 cells) and 64% (204 cells) of ciliated respiratory cells (S9 Fig). The proximal localization pattern of DNAI1 and DNAI2 in respiratory cilia resulted to be characteristic also in *DNAAF7-*mutant individuals OP-6 II5 and OP-596 II2. By quantifying the immunofluorescence analysis in these two individuals we determined that 93% (193 cells) and 90% (205 cells) of cells retained a DNAI1 localization in the proximal ciliary length, respectively for OP-6 II5 and OP-596 II2 (S8 Fig). DNAI2 localization resulted to be proximal in 91% (213 cells) and 90% (151 cells) of ciliated respiratory cells, respectively for OP-6 II5 and OP-596 II2 (S9 Fig). By contrast, *DNAAF4-*mutant (OP-3399), *DNAAF6-*mutant (OP-3141 and OP-1899 II1), *CFAP300-*mutant (OP-2334) and *LRRC6-*mutant (OP-516) individuals presented 100% of ciliated respiratory cells without immunoreactivity against DNAI1 (S8 Fig) and DNAI2 (S9 Fig).

Measurements of the intensity profiles along the flagellar and ciliary axonemes for the DNAI1 and DNAI2 signal additionally confirmed our observations from the quantification analysis. Flagellar axonemes of all dynein preassembly mutant individuals, display a DNAI1 and DNAI2 intensity profile near the baseline value, when compared to control sperm, with single intensity peaks of reduced intensity (S10 and S18 Figs). This shows that DNAI1 and DNAI2 are absent or severely reduced in all studied dynein preassembly mutant individuals.

The intensity profiles along the ciliary axonemes of *DNAAF2*- (OP-146 II3, OP-234 II1) and *DNAAF7*- (OP-6 II5, OP-596 II2) mutant individuals, show for both DNAI1 and DNAI2, either the absence/ severe reduction along the entire ciliary length, or a severe reduction in the distal ciliary length, with its retention in only the proximal part of cilia. The proximal signal intensity of DNAI1 was either comparable to control or reduced of 60–90%, whereas proximal DNAI2 signal intensity resulted to be comparable to control (S11–S13 and S19–S21 Figs). In all other mutant individuals, both DNAI1 and DNAI2 were either absent or severely reduced along ciliary axonemes of respiratory cells (S14–S17 and S22–S25 Figs).

**Fig 7:**
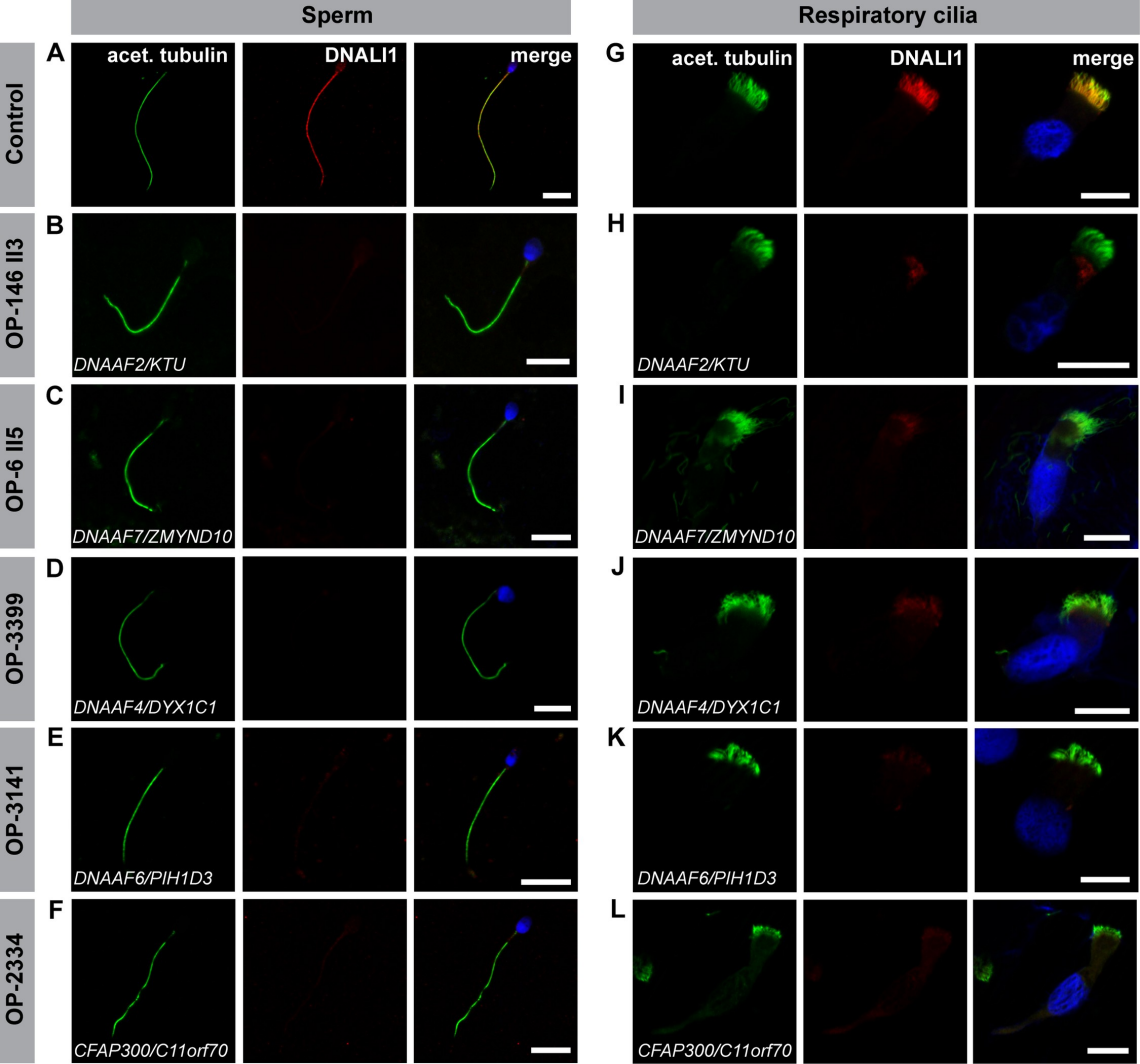
Dynein preassembly mutant sperm flagella and respiratory cilia show absence of the IDA intermediate light chain DNALI1. Control **(A)** and mutant **(B-F)** sperm, as well as control **(G)** and mutant **(H-L)** respiratory cells were double-labeled with antibodies directed against acetylated α tubulin (green) and the inner dynein arm intermediate light chain DNALI1 (red). Both antibodies co-localize along the flagella and cilia in cells from the unaffected control (yellow, **A, G**). In all mutant sperm flagella (**B-F**) and respiratory cells (**H-L**) DNALI1 is absent or severely reduced from the flagellar and ciliary axonemes. Nuclei are stained with Hoechst33342 (blue). Scale bars represent 10 μm.

Due to the difficulties in properly visualizing IDAs by classical structural analyses, such as TEM, we next immuno-labeled sperm cells and nasal brush biopsies from these dynein preassembly mutant individuals against the axonemal dynein intermediate light chain DNALI1, a component of several IDA subtypes [88]. Remarkably, all dynein preassembly-mutant individuals presented here (Table 2) displayed a severe reduction or complete absence of the IDA component DNALI1, in both sperm flagella and respiratory cilia (Figs 7 and S26).

### Structural analyses confirm the outer dynein arm defect in both sperm flagella and respiratory cilia

**Fig 8:**
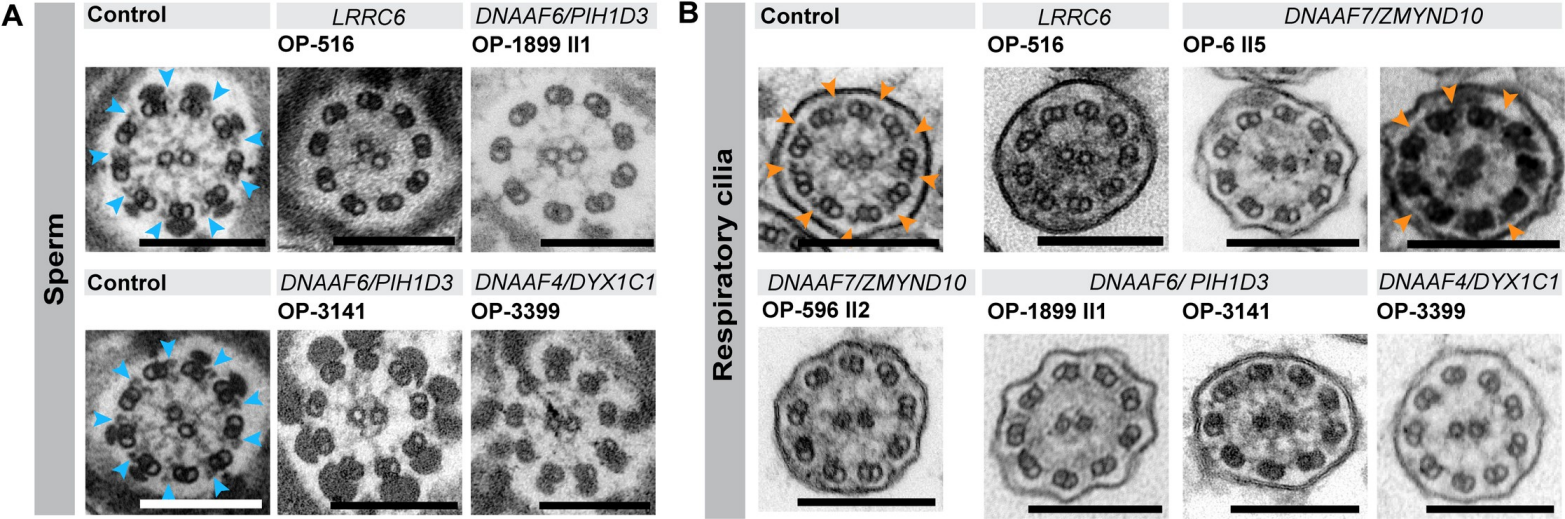
Transmission electron microscopy (TEM) analysis confirms outer dynein arm (ODA) defects in sperm flagella and respiratory cilia from dynein preassembly mutant individuals. **(A)** Electron micrographs of healthy control sperm show a regular flagellar 9+2 axoneme with clearly visible ODAs (indicated by blue arrowheads). Cross sections of sperm flagella of PCD-affected individuals (data shown for OP-516, OP-1899 II1, OP-3141 and OP-3399) confirm the absence and thus the ODA defect identified by high-resolution IF microscopy. **(B)** Analogously, axonemes of respiratory cilia of healthy control individuals show clearly visible and regularly disposed ODAs (indicated by orange arrowheads), whereas those of affected individuals lack the ODA complexes at their outer microtubule doublets. *DNAAF7*-mutant individual OP-6 II5 also displays some ciliary cross sections with ODAs still attached to microtubule doublets. Scale bars represent 200 nm.

To evaluate the ultrastructural defects observed in the mutant sperm and respiratory cells, we additionally performed TEM analyses. Sperm flagellar cross sections were evaluated along the midpiece/ principal piece axis. In respiratory cilia, only cross sections with a definite 9+2 structure, were evaluated. At least 50 cross sections were analyzed per group and cell type. Sperm flagella of healthy control individuals presented a regular 9+2 structure of the axoneme with clearly visible ODAs (Fig 8A).

By contrast, sperm flagella of all PCD-affected individuals presented here (data shown for OP-516, OP-1899 II1, OP-3141 and OP-3399) did not present ODA complexes (Fig 8A). Identical to sperm, transmission-electron micrographs of healthy control respiratory cilia show regularly disposed ODAs at the outer microtubule doublets, whereas those from affected PCD individuals displayed defects of ODAs (Fig 8B). In *DNAAF7*-mutant individual OP-6 II5 we could also observe cross sections with ODAs still attached to some outer microtubule doublets (Fig 8B). These results confirm at the ultrastructural level our findings obtained by high-resolution IF microscopy.

### Dynein preassembly mutant individuals display shorter flagella compared to control sperm from healthy individuals

Previous studies in the green algae *Chlamydomonas* demonstrated that in some dynein preassembly mutant strains flagella appeared to be stumpy or shorter in length [41,75].

**Fig 9:**
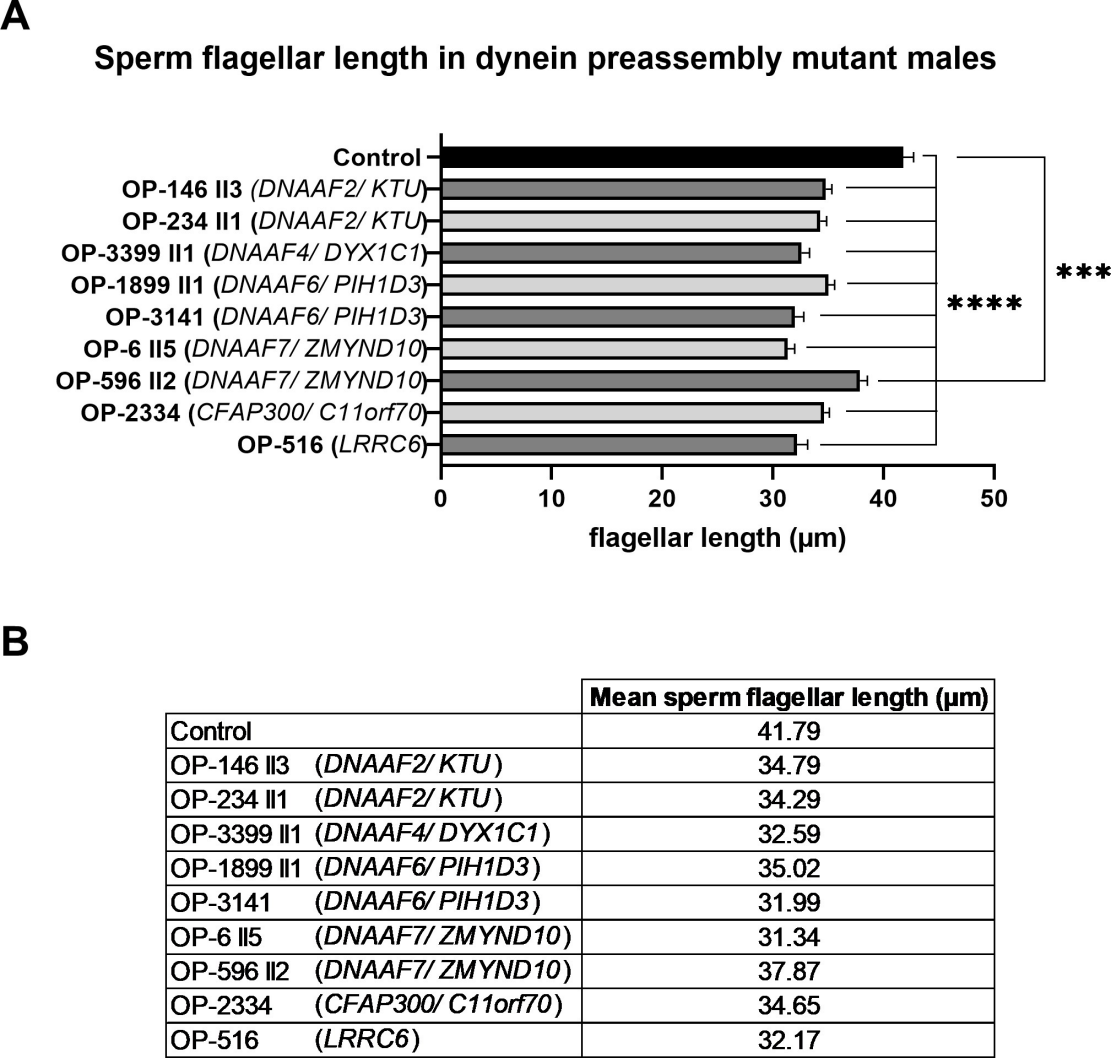
The length of sperm flagella is reduced in dynein preassembly mutant sperm cells. **(A)** Compared to control, sperm flagellar length is significantly reduced in *DNAAF2-* (OP-146 II3 and OP-234 II1), *DNAAF4-* (OP-3399), *DNAAF6-* (OP-1899 II1 and OP-3141), *DNAAF7-* (OP-6 II5 and OP-596 II2), *CFAP300-* (OP-2334) and *LRRC6-* (OP-516) mutant individuals. Statistical analysis was performed using a Brown-Forsythe ANOVA test on the entire dataset (p-value <0.0001: ****) and a two tailed t-test with Welch’s correction comparing each mutant individual to the control group (OP-596 II2 *versus* control: p-value 0.0009 (***); other mutant individuals *versus* control: p-value <0.0001 (****)). **(B)** Table with average value of sperm flagellar length for control sperm and the dynein preassembly mutant individuals analyzed. Error bars represent standard error mean (SEM).

Dynein preassembly mutant individuals OP-516 (*LRRC6*), OP-3141 (*DNAAF6*) and OP-3399 (*DNAAF4*) underwent andrological examination and semen analyses fulfilling the latest WHO guidelines [89]. This included the evaluation of sperm morphology via Papanicolaou staining of sperm samples and the assessment of morphological defects. Interestingly, in those individuals the percentage of sperm with abnormal morphology was slightly increased. We therefore measured the length of sperm flagella from these individuals. Interestingly, in all dynein preassembly mutant individuals, we observed a statistically significant (p-value <0.0001: **** and p-value 0.0009: ***) reduction of the sperm flagellar length (reduction of at least 4 μm) when compared to control sperm (Fig 9 and S2 Appendix).

## Discussion

Here we report a detailed and systematic analysis of the ODA and IDA composition of sperm flagella and respiratory cilia from male PCD individuals carrying disease causing mutations in genes encoding DNAAFs. In total, we studied nine individuals carrying mutations in six (*DNAAF2*, *DNAAF4*, *DNAAF6*, *DNAAF7*, *CFAP300* and *LRRC6*) of the currently twelve reported PCD-associated dynein preassembly genes. Consistent with previous findings, examination of the ciliary motor protein composition by high-resolution IF microscopy in both sperm flagella and respiratory cilia identified defects in proteins known to be crucial in driving flagellar and ciliary motion (ODA components, DNAI1, DNAI2, DNAH8 and DNAH17) and regulating the beat pattern (IDA component, DNALI1) (Figs 4–7, S3–S5 and S26, [18,23,29,50,79]). Additionally, we confirmed the ODA defect at a structural level by TEM (Fig 8). Most motile eukaryotic cilia are built at the cell surface forming distinct hair-like compartments. During this compartmentalized ciliogenesis, axonemal components are transported to the growing cilium through the dynamic mechanism of intraflagellar transport (IFT) [90,91]. However, some ciliated protists and metazoan spermatozoa form their ciliary/flagellar axonemes while exposed to the cytosol in an IFT-independent process [92,93]. It has been speculated that unlike compartmentalized ciliogenesis the formation of mammalian sperm cell flagella could occur in a cytosolic manner, as observed for sperm of the fruit fly *Drosophila melanogaster* [90,93]. However, emerging studies on spermatogenesis in mouse testes have shown that the mammalian sperm tail development indeed requires IFT [94–96], and that the first steps of mammalian flagellogenesis are indistinguishable from those of the compartmentalized ciliogenesis of motile cilia [92,97,98]. Our findings in *DNAAF*-mutant sperm cells support this view.

The dynein preassembly factors examined (DNAAF2, DNAAF4, DNAAF6, DNAAF7, CFAP300 and LRRC6) show high functional conservation from the unicellular green algae *Chlamydomonas* to vertebrates, including fish, mouse and humans. As shown in multiciliated cells of *Xenopus* embryos, dynein axonemal assembly factors form liquid-like organelles in association with axonemal dynein subunits and chaperones. Disruption of DNAAF function results in defective assembly and altered liquid like character of these organelles [99]. Consistent to these findings, dysfunction of DNAAF2, DNAAF4, DNAAF6, DNAAF7, CFAP300 and LRRC6 result in partial or complete absence of ODAs and IDAs in flagella and cilia of several model organisms [23,30,42–44,49–51,53,54,56,57,59–61,64,100].

Currently, two mechanisms are proposed to describe the cytoplasmic preassembly process of dynein arms. Horani *et al*. [48] concluded based on mRNA expression and tagged protein studies in primary airway cell cultures and induced pluripotent stem cell cultures that there is an early preassembly complex consisting of DNAAF5, DNAAF2 and SPAG1 and a late preassembly complex containing DNAAF1, DNAAF3, DNAAF4, LRRC6, DNAAF7 and DNAAF6 [48]. In contrast, Mali *et al*. [56] concluded based on mouse genetic, imaging and quantitative proteomic studies that dynein HCs first interact with the DNAAF7/LRRC6 complex and transfer to HSP90. This complex combines with ICs through interaction with the R2TP complex and with complexes involving interaction of SPAG1 with DNAAF4 and of DNAAF2 with DNAAF6 [56]. Interestingly, while sperm cells of all analyzed DNAAF-deficient males display a severe reduction or the absence of ODAs along their flagella, respiratory cilia of *DNAAF2-* (OP-143 II3 and OP-234 II1) and *DNAAF7-*mutant individuals (OP-6 II5 and OP-596 II2) show preservation of the ODA type 1 (Figs 4, 5, S3, S4, S8, S9, S11–S13 and S19–S21). Based on Horani’s hypothesis, DNAAF2 is supposed to play a role in an early step of cytoplasmic dynein preassembly, whereas DNAAF7 is supposed to function during a later step [48]. According to this model, we would expect that defects in both DNAAFs would cause different phenotypes. However, in both cases, our results show identical cellular defects with preservation of ODA type 1 components in respiratory cilia.

Whitfield and colleagues [18] have shown that sperm flagella present one single ODA type, specifically containing the ODA heavy chains DNAH8 and DNAH17. Since only one distinct ODA type is present in sperm, it is very likely that the disruption of the cytoplasmic preassembly of axonemal dyneins leads to the complete absence of ODAs in sperm. Indeed, flagella of the dynein preassembly-mutant sperm display absence or severe reduction of the sperm specific ODA heavy chains DNAH8 and DNAH17 (Figs 6 and S5).

Although cell type-specific ODA components are present in sperm and respiratory cilia, the phenotypes observed from dynein preassembly mutants described here indicate they are involved in the assembly of these ODA components in both cell types. It is known that dynein arm proteins, such as DNAH5, DNAH9, DNAI1, DNAI2 and DNALI1 are not transported into cilia and sperm flagella if cytoplasmic preassembly of dynein arms is defective. Furthermore, these proteins are not detectable due to rapid degradation of unassembled ODA proteins [19,23,29,50].

We show for the first time that lack of certain dynein axonemal assembly factors causes absence of the sperm specific ODA HCs DNAH8 and DNAH17 from the sperm flagellar axoneme. Consistently, the ODA intermediate chains DNAI1 and DNAI2 are missing, showing that the assembly of ODAs is defective in the analyzed mutant sperms. This indicates that the cytoplasmic dynein preassembly process is highly conserved and biologically relevant also in sperm. However, because the ODA composition of sperm flagella and respiratory cilia differ, genetic defects such as mutations in *DNAAF2* and *DNAAF7* probably result in cell-type specific ciliary/flagellar defects.

Another interesting finding is the significant reduction of sperm flagellar length in dynein preassembly mutant males (Fig 9). This is consistent with similar observations in *Chlamydomonas*. It is reported that in the unicellular alga a lack of multiple axonemal dyneins often results in shortened flagella [41,42,75,101]. The presence of ODAs and IDAs seem to be critical during flagellogenesis or for the stability of axonemal microtubules. In line with this observation, biallelic mutations in *DNAH8* and *DNAH17*, as well as *DNAH1*, not only cause ODA or IDA defects in sperm, but also multiple morphological abnormalities of the sperm flagella (MMAF), a severe form of asthenoteratozoospermia with highly genetic heterogeneity [18,102,103]. In this study, semen analyses of three affected individuals displayed severe morphological sperm abnormalities in accordance with current WHO criteria (lower reference limit for normal form: 4%) [89] (S1 Appendix). Due to the low percentage of normal sperm cells in all three investigated males (none with > 4% normal morphology) in addition to the significantly shorter sperm flagella, we assume, that individuals with defective dynein preassembly could also display a MMAF phenotype. Future studies in distinct dynein preassembly mutants are needed to elucidate this important point in more detail.

PCD is a motile ciliopathy also exhibiting remarkable genetic heterogeneity [24]. Currently, mutations in more than 50 different genes are linked to PCD [24]. Affected individuals mainly suffer from recurrent infections of the upper and lower respiratory tract. Because motile respiratory cilia and sperm flagella have common axonemal structures, it is assumed that most men affected by PCD are infertile [104]. However, spontaneous paternity in PCD patients has been reported. A genotype-phenotype study to assess the fertility status in an adult cohort of PCD individuals by Vanaken and colleagues [105] demonstrated that PCD individuals with combined ODA and IDA defects or IDA defects with microtubule disorganization in respiratory cilia are more likely to be infertile, than PCD individuals with defects in respiratory cilia of other categories, such as isolated ODA defects or radial spoke defects [105]. These data support our observation that the cytoplasmic dynein preassembly machinery is highly conserved in both sperm flagella and respiratory cilia.

Our findings also highlight the utility of employing alternative techniques for TEM to analyze axonemal composition of sperm flagella and respiratory cilia. TEM is largely limited to the detection of ODA defects or microtubular disorganization [106]. High-resolution IF analysis allows the molecular characterization of specific axonemal components that are difficult to detect by TEM, such as IDAs (e.g. DNALI1), and this technique can reveal cell-specific differences including differential subcellular distribution (e.g. DNAI1). This study provides an additional example of using high-resolution IF microscopy to assess the impact of disease-causing mutations on axonemal protein composition in different cell types.

One limitation of our study is that it does not address the full spectrum of disease-causing dynein preassembly mutations. Five other dynein preassembly genes (*DNAAF1*, *DNAAF3*, *DNAAF5*, *SPAG1*, and *CFAP298* (*C21orf59*)) are known to be associated with PCD and putatively, male infertility [26,34,35,41,47,65,79]. While it is reasonable to speculate that mutations in these other PCD-associated genes involved in dynein preassembly would cause combined ODA and IDA defects in both sperm flagella and respiratory cilia, a detailed analysis of the fertility status in affected males and comparison of the dynein arm composition between sperm flagella and respiratory cilia is required. Very recently, *TTC12* loss-of-function mutations were identified to cause combined ODA and IDA defects in sperm flagella but only the absence of IDAs in respiratory cilia, unveiling an additional factor that distinguishes dynein assembly mechanisms in sperm flagella from respiratory cilia [68]. Further analyses of males affected by mutations in *DNAAF1*, *DNAAF3*, *DNAAF5*, *SPAG1*, and *CFAP298* (*C21orf59*)) will provide a more comprehensive picture of the dynein preassembly process and possibly reveal additional differences between these cell types.

In conclusion, our results show that disruption of the cytoplasmic dynein preassembly process leads to a defect of dynein arms in sperm flagella as well as respiratory cilia, demonstrating that the process of cytoplasmic dynein preassembly is highly conserved and also critical in the development of the mammalian sperm flagellum. However, distinct dynein assembly mechanisms between these cell types are also possible [68].

While additional studies on PCD individuals carrying mutations in other dynein preassembly genes are warranted, this study provides a first step in describing the sperm phenotypes of distinct dynein preassembly mutations, but also raises the awareness of important further disease manifestations, such as infertility, in PCD individuals. Our findings therefore will enhance the clinical and molecular understanding of distinct PCD-variants. Translation of study results into patient care will improve genetic and andrological counselling of PCD individuals and their families and probably facilitate treatment strategies in case of assisted reproduction.

## Materials and methods

### Ethic statement

This research project was approved by the ethics committee from the Medical Association Westphalia Lippe and the Westphalian Wilhelms University Münster (Ärztekammer Westfalen Lippe und Westfälische Wilhelms Universität). The ethics committee declared to have no fundamental ethical or legal objections against the implementation of the research project (reference number: 2017-139-f-s). Signed and informed consent was obtained from all participating individuals fulfilling diagnostic criteria and from family members.

### Study cohort

In this study, eight adult males diagnosed with PCD, due to disease causing mutations in genes encoding DNAAFs, were retrospectively and prospectively examined for their fertility status and sperm flagella function. When applicable, individuals underwent andrological examination, including ultrasound of the testes, and hormone and semen analyses at the Centre for Reproductive Medicine and Andrology (CeRA), University Hospital, Muenster (Germany). Moreover, by systematically investigating their ODA and IDA composition in sperm flagella and respiratory cilia, the purpose was to compare on a functional level the dynein preassembly process between these two distinct cell types. PCD individuals for this study were selected from our patient cohort based on their gender, age (at least 18 years old) and mutational status.

Signed and informed consent was obtained from individuals fulfilling diagnostic criteria and from family members according to protocols approved by the institutional ethics review board at the University of Muenster (Muenster, Germany) and collaborating institutions.

### Human samples

Ejaculate samples and respiratory cells from nasal brush biopsies were collected from eight PCD and healthy control individuals. Nasal brush biopsies and semen (see subheading “semen analysis”) of each individual were used for high-resolution immunofluorescence microscopy (IF) and high-speed video microscopy analyses (HVMA). When possible, transmission electron microscopy (TEM) was performed on both cell types for ultrastructural analyses (Table 2).

### Semen analysis

Semen analysis was conducted in the accredited andrology laboratory of the Centre for Reproductive Medicine and Andrology (CeRA), University Hospital, Münster (Germany), fulfilling latest WHO guidelines [89] and was under constant internal and external quality control. Semen of healthy volunteers and recruited PCD individuals were collected by masturbation after 2–7 days of sexual abstinence and evaluated after liquefaction for 30 min at 37°C to assess the sperm concentration and motility, as well as the vitality and morphology. Remaining ejaculate of each individual was used for IF, TEM and HVMA.

### Mutational analysis

Mutational analysis in PCD affected individuals with combined ODA and IDA defects of unknown genetic cause was performed using whole-exome sequencing (WES) and Sanger sequencing (OP-6 II5, OP-596 II2, OP-3141; primer sequences available on request), as previously described [17,29,50]. Variants present in the dbSNP database, the1000 Genomes Project polymorphism, and the Genome Aggregation Database (gnomAD) with a minor-allele frequency >0.01 percent were excluded. We further selected for nonsynonymous mutations, splice-site substitutions, and indels following an autosomal- and X-linked recessive inheritance pattern. Furthermore, for OP-3399, a customized PCD gene panel comprising 33 genes (S3 Table) for targeted next generation sequencing (NGS) was carried out at the Institute of Human Genetics, Muenster, within the Male Reproductive Genomics (MERGE) Study. This targeted gene panel was enriched using a customized Agilent’s SureSelectQXT Target Enrichment kit. For multiplexed sequencing, the libraries were index tagged using appropriate pairs of index primers. Quantity and quality of the libraries were measured with the ThermoFisher Qubit and Agilent's TapeStation 2200, respectively. Sequencing was conducted on an Illumina NextSeq500 using NextSeq500 V2 High-Output kit. After trimming and removing the remaining adapter sequences (Cutadapt v1.15), alignment of sequence reads to the reference genome GRCh37.p13 (BWA Mem v0.7.17), and calling of variants (GATK toolkit v3.8 with HaplotypeCaller, in accordance with the best practice recommendations), Ensembl Variant Effect Predictor was used for annotation. Variant filtering and assessment of transcript and functional consequences, population frequencies, and *in silico* predicted relevance were carried out utilizing the Institute of Human Genetics’ in-house pipeline Sciobase. Genomic DNA was isolated by standard methods directly from blood samples.

### High-speed video microscopy analyses (HVMA)

To assess motility of sperm cells, 10 μl of liquefied human ejaculate was analyzed at 20x and 40x magnification with an Eclipse Ti Inverted Microscope (Nikon, Tokyo, Japan) connected to an acA1300-200um—Basler ace camera (Basler AG, Ahrensburg, Germany) at 37°C. Using the same instrumentation, cilia beat frequency and pattern was analyzed at 40 x and 63 x magnification at 25°C. Video analyses were performed with the Sisson-Ammons Video Analysis (SAVA) system (Ammons Engineering, Clio, USA) and exported videos processed with VideoMach 2.7.2 Software (http://gromada.com/main/products.php), as described before [107].

### High-resolution immunofluorescence (IF) microscopy

Human sperm cells were analyzed after ejaculate donation and multiciliated respiratory cells after nasal brush sampling. Human cell samples were treated and incubated by primary and secondary antibodies as reported previously [108]. As marker for the ciliary or flagellar axoneme, monoclonal mouse anti-acetylated α-tubulin antibody (Sigma-Aldrich, Taufkirchen, Germany) was used. Polyclonal rabbit antibodies directed against DNAI1, DNAI2, and DNALI1 were reported previously [50]. Polyclonal rabbit antibodies directed against DNAH8 (HPA028447) and DNAH17 (HPA024354) were purchased respectively from Sigma Prestige Antibodies and Atlas Antibodies. For immunofluorescence analysis on sperm the anti-DNAH8 antibody was diluted 1:250, whereas anti-DNAH17 antibody was diluted 1:200. Goat anti-mouse Alexa Fluor 488, as well as goat anti-rabbit Alexa Fluor 546 (Thermo Fischer Scientific, Waltham, USA) were used as secondary antibodies. Nuclei were stained with DAPI or Hoechst33342 (Thermo Fischer Scientific, Waltham, USA) and all samples were mounted in fluorescence mounting medium (Dako North America Inc., Carpinteria, USA). Immunofluorescence (IF) images were captured with a Zeiss Laser Scanning Microscope LSM 880 (Carl Zeiss Microscopy GmbH, Jena, Germany). Pictures were processed with ZEISS ZEN Imaging Software 2012 (Carl Zeiss Microscopy GmbH, Jena, Germany) and Adobe Creative Suite CS5 (Adobe Systems, San José, USA). Measurement of signal intensity was performed on sperm flagella, ciliated respiratory cilia and single respiratory cilia in at least two different experiments, using ZEISS ZEN Blue imaging software 2012 (Carl Zeiss Microscopy GmbH, Jena, Germany).

### Transmission electron microscopy (TEM)

Human respiratory cells obtained from nasal brush biopsies and 250 μl of liquefied ejaculates were suspended each in 2.5% glutaraldehyde for fixation. Samples were processed for TEM analysis according to standardized protocols [109]. Pelleted human cell samples were contrasted with 1% osmium tetroxide, incubated in a 1, 2-epoxypropan-epon mixture (1:2) at 4°C overnight and finally embedded in epon. After polymerization, semithin sections were first cut from all samples and stained by tolouidine blue. Afterwards ultrathin sections (80 nm) were prepared and collected onto copper grids for TEM analyses. Samples were analyzed with the transmission electron microscope Philips CM10 and TEM images acquired with a Quemesa camera and the iTEM SIS image acquisition software (both from Olympus Soft Imaging Solutions). Image processing was performed with Adobe Creative Suite CS5.

### Nasal nitric oxide

Measurement of nasal nitric oxid (nNO)-production rate as part of the routine diagnostic for PCD was performed as previously described [110]. In this study a chemiluminescence analyzer was used per current guidelines for PCD diagnostic [80]. Patients have to perform a velum closure technique. nNO production-rate is low in most PCD variants. A nNO-production rate of less than 77 nL/min is the established cutoff for PCD referral cases [81].

### Statistical analyses

Statistical analysis was performed using GraphPad PRISM 8.0 (GraphPad Software, San Diego, USA). For the sperm flagellar length measurements, the entire dataset was analyzed via a Brown-Forsythe ANOVA test for group analysis. Statistical comparison of each mutant individual to the control group was performed via a two tailed t-test with Welch’s correction. Graphical representation of quantifications of the immunofluorescence analyses and of sperm flagellar length was performed using the same software.

## Supporting information

S1 Fig Family pedigrees of PCD affected individuals with combined ODA/IDA defects. (PDF)

S2 Fig Genetic testing in PCD-affected infertile individual OP-516 identifies diseases causing mutations in the gene *LRRC6*, encoding a dynein axonemal assembly factor. (PDF)

S3 Fig Mutant sperm flagella and respiratory cilia show absence or reduction of the ODA intermediate chain DNAI1. (PDF)

S4 Fig Mutant sperm flagella and respiratory cilia show absence or reduction of the ODA intermediate chain DNAI2. (PDF)

S5 Fig Mutant sperm flagella show absence of the sperm specific ODA heavy chains DNAH8 and DNAH17. (PDF)

S6 Fig Quantification of DNAI1 immunofluorescence (IF) in sperm of healthy control and dynein preassembly mutant individuals. (PDF)

S7 Fig Quantification of DNAI2 immunofluorescence (IF) in sperm of healthy control and dynein preassembly mutant individuals. (PDF)

S8 Fig Quantification of DNAI1 immunofluorescence (IF) in respiratory cells of healthy control and dynein preassembly mutant individuals. (PDF)

S9 Fig Quantification of DNAI2 immunofluorescence (IF) in respiratory cells of healthy control and dynein preassembly mutant individuals. (PDF)

S10 Fig Measurement of the DNAI1 fluorescence intensity along the flagellar axonemes of control and dynein preassembly mutant sperm. (PDF)

S11 Fig Measurement of the DNAI1 fluorescence intensity along the ciliary axonemes of control respiratory cells. (PDF)

S12 Fig Measurement of the DNAI1 fluorescence intensity along the ciliary axonemes of *DNAAF2/KTU*-mutant respiratory cells. (PDF)

S13 Fig Measurement of the DNAI1 fluorescence intensity along the ciliary axonemes of *DNAAF7/ZMYND10*-mutant respiratory cells. (PDF)

S14 Fig Measurement of the DNAI1 fluorescence intensity along the ciliary axonemes of *DNAAF4/DYX1C1*-mutant respiratory cells. (PDF)

S15 Fig Measurement of the DNAI1 fluorescence intensity along the ciliary axonemes of *DNAAF6/PIH1D3*-mutant respiratory cells. (PDF)

S16 Fig Measurement of the DNAI1 fluorescence intensity along the ciliary axonemes of *CFAP300/C11orf70*-mutant respiratory cells. (PDF)

S17 Fig Measurement of the DNAI1 fluorescence intensity along the ciliary axonemes of *LRRC6*-mutant respiratory cells. (PDF)

S18 Fig Measurement of the DNAI2 fluorescence intensity along the flagellar axonemes of control and dynein preassembly mutant sperm. (PDF)

S19 Fig Measurement of the DNAI2 fluorescence intensity along the ciliary axonemes of control respiratory cells. (PDF)

S20 Fig Measurement of the DNAI2 fluorescence intensity along the ciliary axonemes of *DNAAF2/KTU*-mutant respiratory cells. (PDF)

S21 Fig Measurement of the DNAI2 fluorescence intensity along the ciliary axonemes of *DNAAF7/ZMYND10*-mutant respiratory cells. (PDF)

S22 Fig Measurement of the DNAI2 fluorescence intensity along the ciliary axonemes of *DNAAF4/DYX1C1*-mutant respiratory cells. (PDF)

S23 Fig Measurement of the DNAI2 fluorescence intensity along the ciliary axonemes of *DNAAF6/PIH1D3*-mutant respiratory cells. (PDF)

S24 Fig Measurement of the DNAI2 fluorescence intensity along the ciliary axonemes of *CFAP300/C11orf70*-mutant respiratory cells. (PDF)

S25 Fig Measurement of the DNAI2 fluorescence intensity along the ciliary axonemes of *LRRC6*-mutant respiratory cells. (PDF)

S26 Fig Mutant sperm flagella and respiratory cilia show absence or reduction of the IDA intermediate chain DNALI1. (PDF)

S1 Table Primer pairs used for amplification of *DNAAF6* exons and DNAI1 exon 3 in OP-3141. (PDF)

S2 Table Primer sequences used for Sanger Sequencing. (PDF)

S3 Table List of 33 genes in customized PCD panel. (PDF)

S1 Video Control ciliated respiratory cells. (MP4)

S2 Video OP-6 II5 ciliated respiratory cell. (MP4)

S3 Video OP-596 II2 ciliated respiratory cell. (MP4)

S4 Video OP-3141 ciliated respiratory cell. (MP4)

S5 Video OP-3399 ciliated respiratory cells. (MP4)

S6 Video OP-516 ciliated respiratory cells. (MP4)

S7 Video Control sperm. (MP4)

S8 Video OP-6 II5 sperm. (MP4)

S9 Video OP-596 II2 sperm. (MP4)

S10 Video OP-3141 sperm. (MP4)

S11 Video OP-3399 sperm. (MP4)

S12 Video OP-516 sperm. (MP4)

S1 Appendix Semen analysis of study participants. (XLSX)

S2 Appendix Dataset and statistical analysis of sperm flagellar length measurement. (XLSX)
