# Supplementary material for: Defects in the cytoplasmic assembly of axonemal dynein arms cause morphological abnormalities and dysmotility in sperm cells leading to male infertility
Source: PLoS Genet. 2021 Feb 26;17(2):e1009306. doi: 10.1371/journal.pgen.1009306 (PMC7909641; doi:10.1371/journal.pgen.1009306)
Supplement: S1 Fig — (PDF) [file pgen.1009306.s001.pdf]

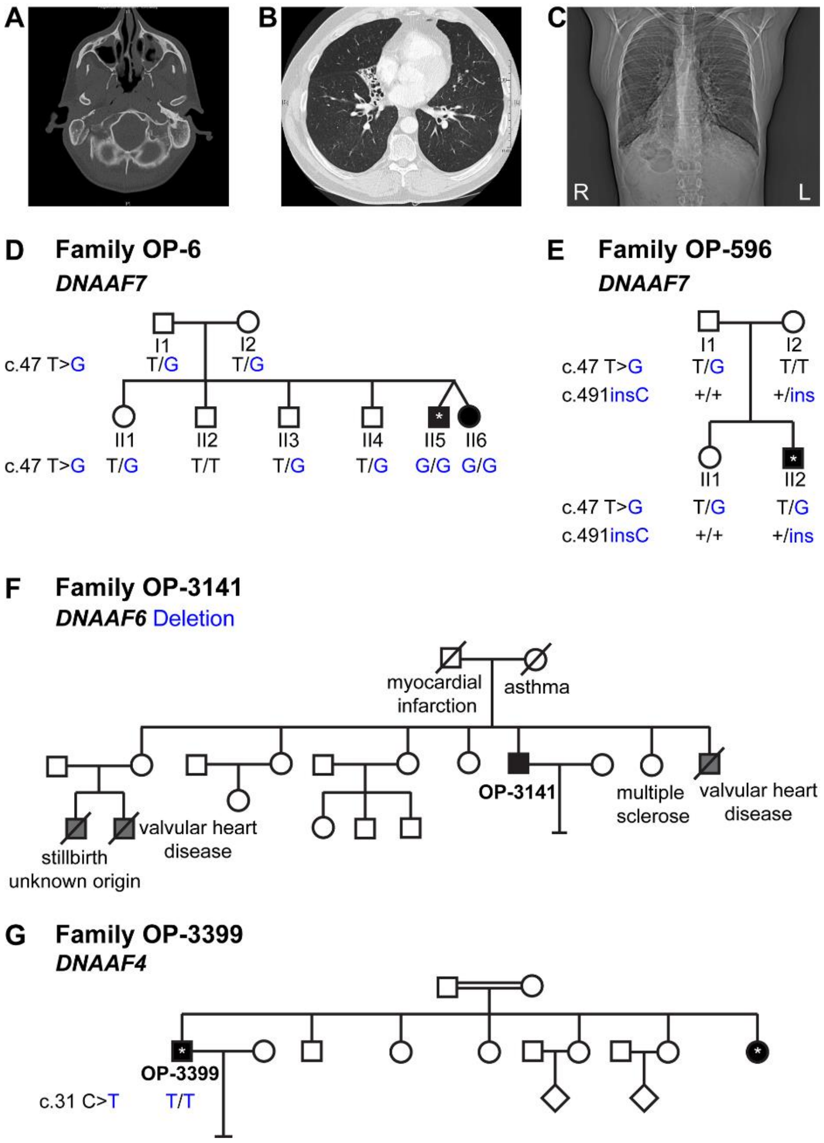

S1 Fig. Family pedigrees of PCD affected individuals with combined ODA/ IDA defects. **(A)** Cranial CT showing chronic sinusitis in OP-1899 II1. **(B)** CT thorax of OP-3141 displaying destructed middle lobe and bronchiectasis. **(C)** Chest X-ray of OP-1899 II1 displaying *situs inversus totalis* (mirror image of thorax and abdominal organs; **R**: right; **L**: left). **(D** and **E)** Pedigree structure and segregation analysis of PCD-affected families harboring *DNAAF7* mutations. **(F)** Pedigree structure of PCD-affected family harboring a hemizygous *DNAAF6* mutation. **(G)** Pedigree structure of PCD-affected family harboring *DNAAF4* mutations. (**D**-**G**) Black filled symbols indicate affected individuals. An asterisk indicates *situs inversus*. The plus sign indicates a normal allele and "ins" stands for insertion. Slash sign indicates death of an individual. Segregation analysis for families OP-3141 and OP-3399 (**C** and **D**) was not possible due to unavailability of parental DNA and/ or genomic material from siblings.
