# Supplementary material for: Defects in the cytoplasmic assembly of axonemal dynein arms cause morphological abnormalities and dysmotility in sperm cells leading to male infertility
Source: PLoS Genet. 2021 Feb 26;17(2):e1009306. doi: 10.1371/journal.pgen.1009306 (PMC7909641; doi:10.1371/journal.pgen.1009306)
Supplement: S2 Fig — (PDF) [file pgen.1009306.s002.pdf]

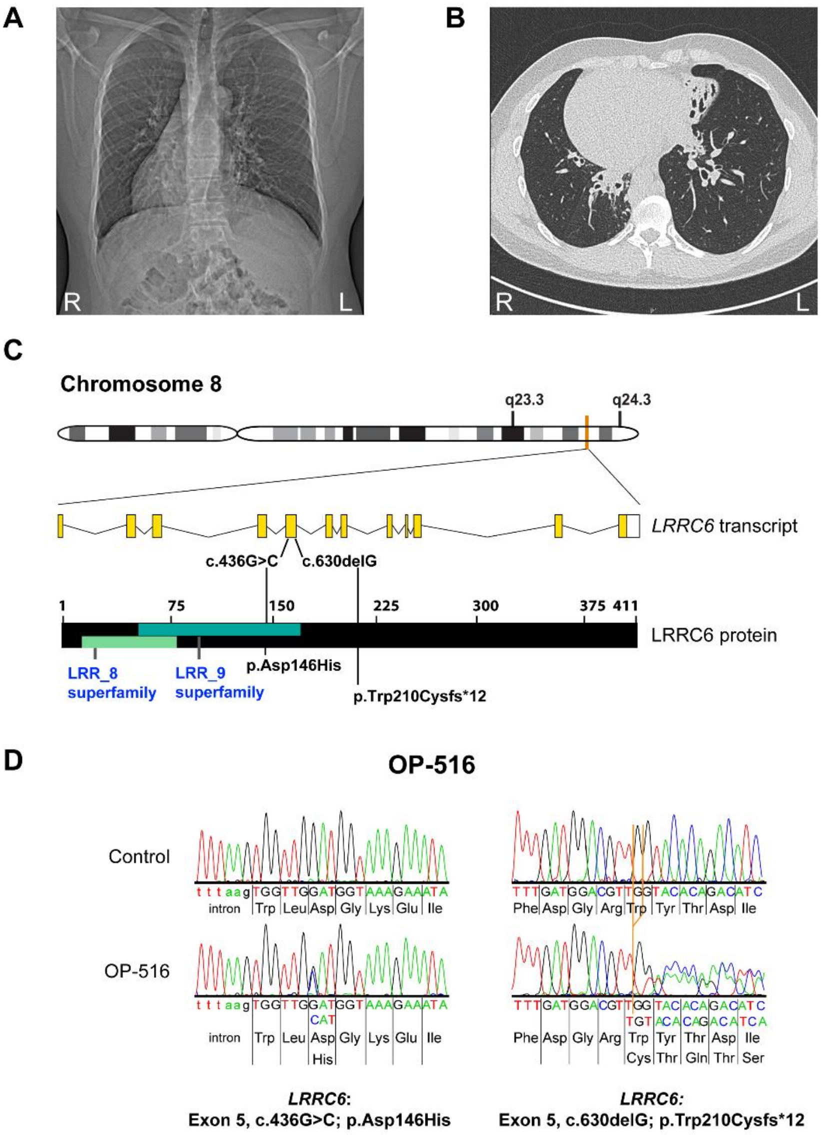

S2 Fig. Genetic testing in PCD-affected infertile individual OP-516 identifies diseases causing mutations in the gene *LRRC6*, encoding a dynein axonemal assembly factor. **(A)** CT thorax (frontal view) of OP-516 displaying *situs inversus totalis* (mirror image of thorax and abdominal organs; R: right; L: left). **(B)** CT thorax (transversal view) of OP-516 displaying bilateral bronchiectasis and destruction of the middle lobe (R: right; L: left). **(C)** *LRRC6* (CCDS6365) is located on chromosome 8q24.22 (orange mark in the chromosome schematic). It consists of twelve coding exons, encoding a 466 amino-acid protein. **(D)** Sanger sequencing for PCD affected individual OP-516 confirmed compound heterozygous mutations in Exon 5 (c.436G>C, p.Asp146His and c.630delG, p.Trp210Cysfs*12).
