# Supplementary material for: Defects in the cytoplasmic assembly of axonemal dynein arms cause morphological abnormalities and dysmotility in sperm cells leading to male infertility
Source: PLoS Genet. 2021 Feb 26;17(2):e1009306. doi: 10.1371/journal.pgen.1009306 (PMC7909641; doi:10.1371/journal.pgen.1009306)
Supplement: S4 Fig — (PDF) [file pgen.1009306.s004.pdf]

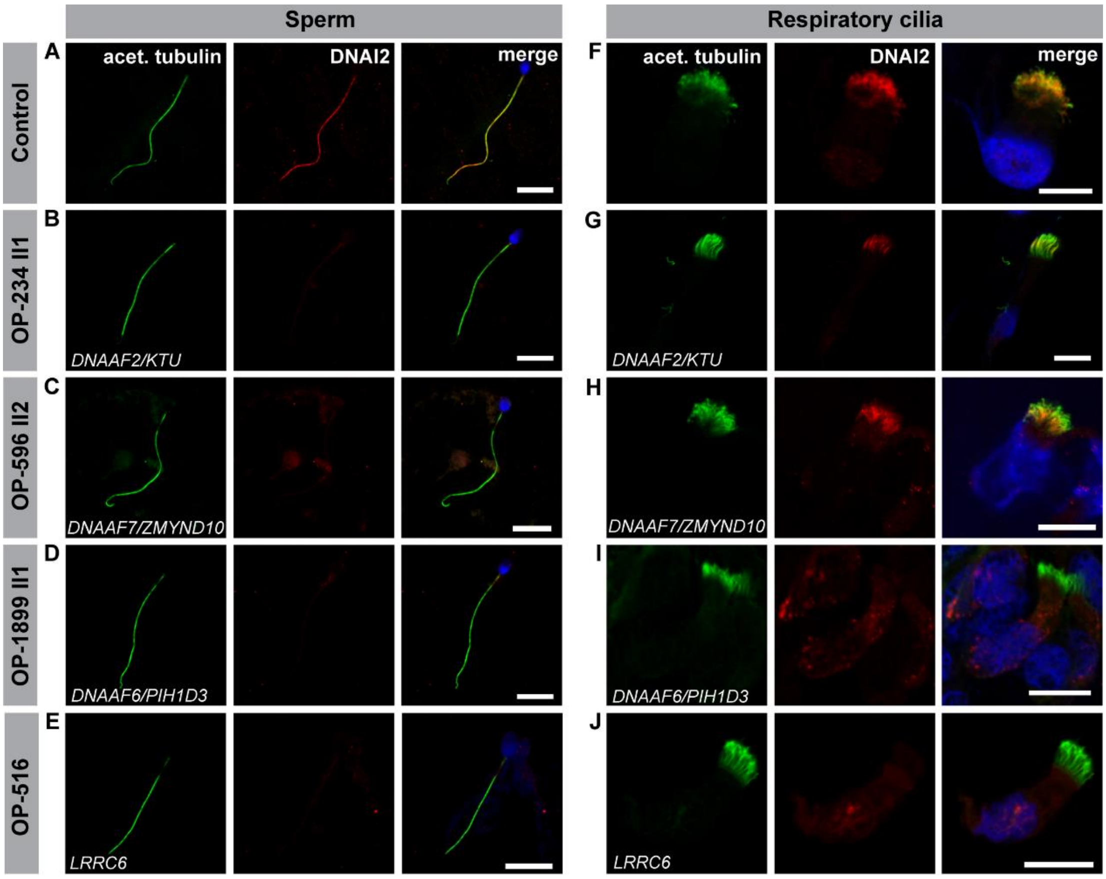

S4 Fig. Mutant sperm flagella and respiratory cilia show absence or reduction of the ODA intermediate chain DNAI2. Control (**A**) and mutant (**B**-**E**) sperm cells, as well as control (**F**) and mutant (**G**-**J**) respiratory cells were double-labeled with antibodies directed against acetylated α tubulin (green) and the outer dynein arm intermediate chain DNAI2 (red). Both antibodies co-localize along the flagella and cilia in cells from the unaffected control (yellow, **A**, **F**). In all mutant sperm cells DNAI2 was not detected along the flagellar axoneme (**B**-**E**). In mutant respiratory cells of OP-234 II1 (**G**) and OP-596 II2 (**H**), DNAI2 localized to the proximal ciliary length, whereas in mutant cells of OP-1899 II1 (**I**) and OP-516 (**J**) DNAI2 was not detected along the ciliary axoneme. Nuclei were stained with Hoechst33342 (blue). Scale bars represent 10 µm.
