# Supplementary material for: Defects in the cytoplasmic assembly of axonemal dynein arms cause morphological abnormalities and dysmotility in sperm cells leading to male infertility
Source: PLoS Genet. 2021 Feb 26;17(2):e1009306. doi: 10.1371/journal.pgen.1009306 (PMC7909641; doi:10.1371/journal.pgen.1009306)
Supplement: S5 Fig — (PDF) [file pgen.1009306.s005.pdf]

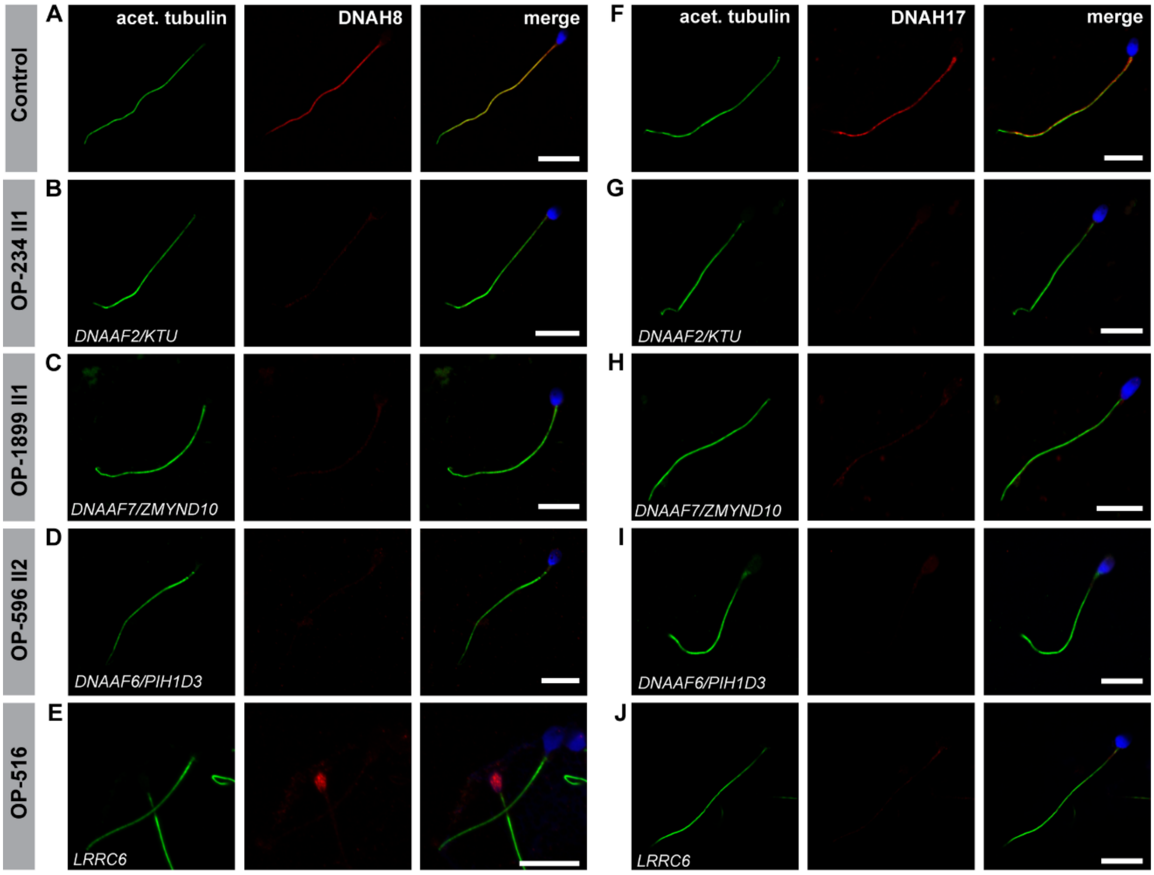

S5 Fig. Mutant sperm flagella show absence of the sperm specific ODA heavy chains DNAH8 and DNAH17. Control (**A** and **F**) and mutant sperm (**B**-**E** and **G**-**J**) were double-labeled with antibodies directed against acetylated α tubulin (green), as flagellar marker, and the sperm specific outer dynein arm heavy chains DNAH8 (**A**-**E**, red) and DNAH17 (**F**-**J**, red). Both antibodies co-localize along the flagella from the unaffected control (merged image, **A**, **F**). In all mutant sperm flagella DNAH8 and DNAH17 were not detected along the flagellar axoneme (**B**-**E**, **G**-**J**). Nuclei were stained with Hoechst33342 (blue). Scale bars represent 10 µm.
