# Supplementary material for: Defects in the cytoplasmic assembly of axonemal dynein arms cause morphological abnormalities and dysmotility in sperm cells leading to male infertility
Source: PLoS Genet. 2021 Feb 26;17(2):e1009306. doi: 10.1371/journal.pgen.1009306 (PMC7909641; doi:10.1371/journal.pgen.1009306)
Supplement: S6 Fig — (PDF) [file pgen.1009306.s006.pdf]

A

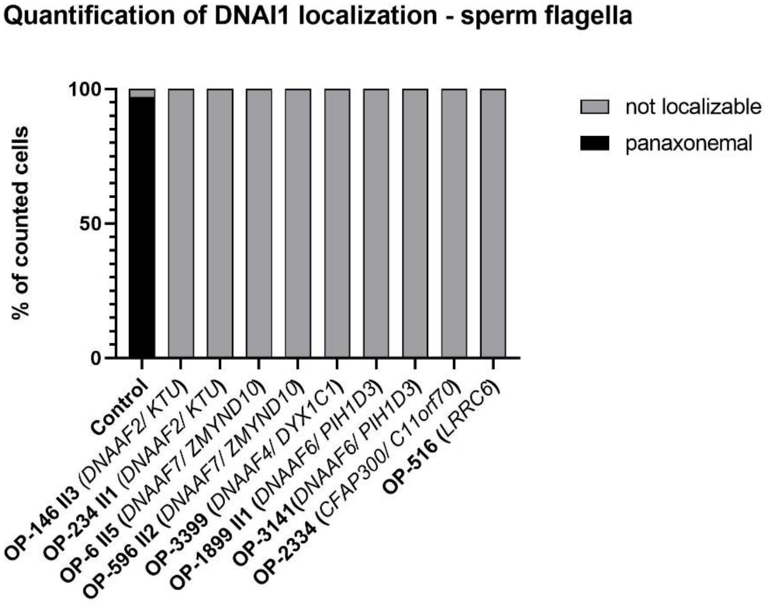

B

	panaxonemal	not localizable	n° counted cells	replicates
**Control**	356	11	367	3
**OP-146 II3 (*DNAAF2/ KTU*)**	0	330	330	3
**OP-234 II1 (*DNAAF2/ KTU*)**	0	292	292	3
**OP-6 II5 (*DNAAF7/ ZMYND10*)**	0	204	204	3
**OP-596 II2 (*DNAAF7/ ZMYND10*)**	0	265	265	3
**OP-3399 (*DNAAF4/ DYX1C1*)**	0	188	188	3
**OP-1899 II1 (*DNAAF6/ PIH1D3*)**	0	276	276	3
**OP-3141 (*DNAAF6/ PIH1D3*)**	0	144	144	3
**OP-2334 (*CFAP300/ C11orf70*)**	0	199	199	3
**OP-516 (*LRRC6*)**	0	279	279	3

S6 Fig. Quantification of DNAI1 immunofluorescence (IF) in sperm of healthy control and dynein preassembly mutant individuals. (A) Graph representing the quantification analysis performed for the DNAI1 IF on sperm. In control sperm 97% of cells display a panaxonemal localization of DNAI1, whereas in 3% of sperm immunoreactivity against DNAI1 is not observed. By contrast, in 100% of sperm cells counted for each dynein preassembly mutant individual, DNAI1 is not localized along the flagellar length. **(B)** Table describing the total number of cells counted per group and localization pattern observed. The quantification analysis for sperm cells represents three independent experiments.
