# Supplementary material for: Defects in the cytoplasmic assembly of axonemal dynein arms cause morphological abnormalities and dysmotility in sperm cells leading to male infertility
Source: PLoS Genet. 2021 Feb 26;17(2):e1009306. doi: 10.1371/journal.pgen.1009306 (PMC7909641; doi:10.1371/journal.pgen.1009306)
Supplement: S7 Fig — (PDF) [file pgen.1009306.s007.pdf]

A

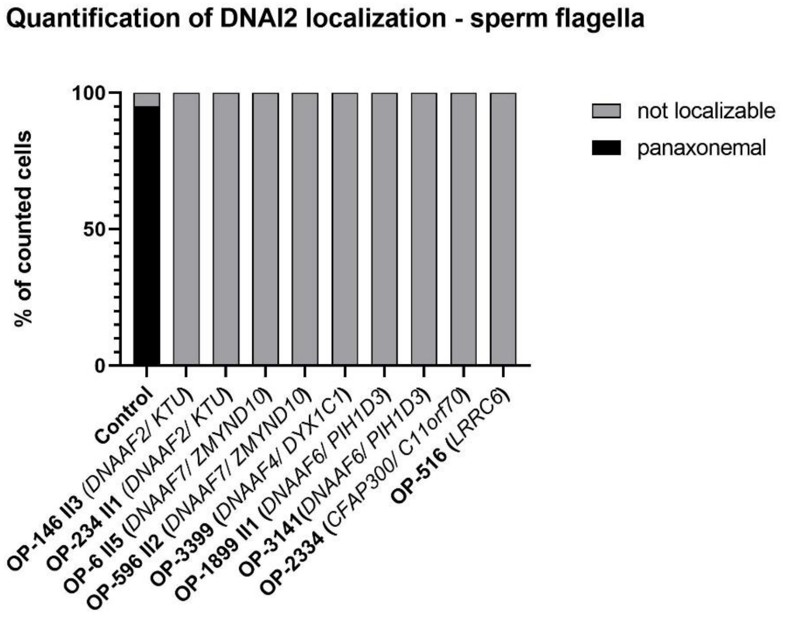

B

	panaxonemal	not localizable	n° counted cells	replicates
**Control**	329	18	347	3
**OP-146 II3 (*DNAAF2/ KTU*)**	0	201	201	3
**OP-234 II1 (*DNAAF2/ KTU*)**	0	271	271	3
**OP-6 II5 (*DNAAF7/ ZMYND10*)**	0	166	166	3
**OP-596 II2 (*DNAAF7/ ZMYND10*)**	0	187	187	3
**OP-3399 (*DNAAF4/ DYX1C1*)**	0	184	184	3
**OP-1899 II1 (*DNAAF6/ PIH1D3*)**	0	250	250	3
**OP-3141 (*DNAAF6/ PIH1D3*)**	0	258	258	3
**OP-2334 (*CFAP300/ C11orf70*)**	0	200	200	3
**OP-516 (*LRRC6*)**	0	246	246	3

S7 Fig. Quantification of DNAI2 immunofluorescence (IF) in sperm of healthy control and dynein preassembly mutant individuals. (A) Graph representing the quantification analysis performed for the DNAI2 IF on sperm. In control sperm 95% of cells display a panaxonemal localization of DNAI1, whereas in 5% of sperm immunoreactivity against DNAI2 is not observed. By contrast, in 100% of sperm cells counted for each dynein preassembly mutant individual, DNAI2 is not localized along the flagellar length. **(B)** Table describing the total number of cells counted per group and localization pattern observed. The quantification analysis for sperm cells represents three independent experiments.
