# Supplementary material for: Defects in the cytoplasmic assembly of axonemal dynein arms cause morphological abnormalities and dysmotility in sperm cells leading to male infertility
Source: PLoS Genet. 2021 Feb 26;17(2):e1009306. doi: 10.1371/journal.pgen.1009306 (PMC7909641; doi:10.1371/journal.pgen.1009306)
Supplement: S8 Fig — (PDF) [file pgen.1009306.s008.pdf]

A

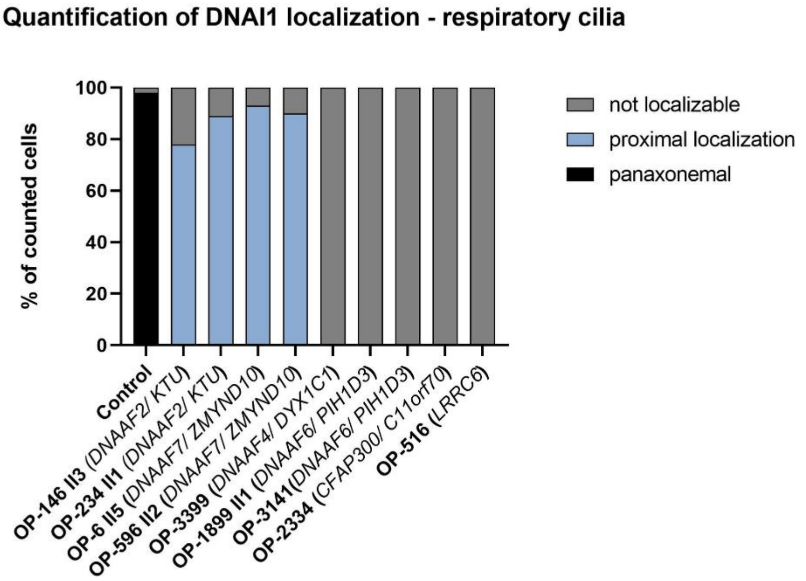

B

	panaxonemal	proximal localization	not localizable	n° counted cells	replicates
**Control**	325	0	7	332	3
**OP-146 II3 (*DNAAF2/ KTU*)**	0	50	14	64	2
**OP-234 II1 (*DNAAF2/ KTU*)**	0	96	12	108	3
**OP-6 II5 (*DNAAF7/ ZMYND10*)**	0	179	14	193	3
**OP-596 II2 (*DNAAF7/ ZMYND10*)**	0	184	21	205	3
**OP-3399 (*DNAAF4/ DYX1C1*)**	0	0	143	143	3
**OP-1899 II1 (*DNAAF6/ PIH1D3*)**	0	0	125	125	3
**OP-3141 (*DNAAF6/ PIH1D3*)**	0	0	72	72	3
**OP-2334 (*CFAP300/ C11orf70*)**	0	0	202	202	3
**OP-516 (*LRRC6*)**	0	0	116	116	3

S8 Fig. Quantification of DNAI1 immunofluorescence (IF) in respiratory cells of healthy control and dynein preassembly mutant individuals. (A) Graph representing the quantification analysis performed for the DNAI1 IF on respiratory cilia. In control nasal brush biopsies, DNAI1 is localized along the entire ciliary length in 98% of cells, whereas in 2% of cells immunoreactivity against DNAI1 is not detected. In the *DNAAF2*-mutant individuals OP-146II3 and OP-243II1, a proximal localization of DNAI1 is observed in 78% and 89% of counted cells, respectively, whereas the remaining cells counted (22% for OP-146 II3 and 11% for OP-234 II1) do not display immunoreactivity against DNAI1. In addition, *DNAAF7*-mutant individuals OP-6 II5 and OP-596 II2 show a proximal localization pattern for DNAI1 in 93% and 90% of their respiratory cilia, respectively (no DNAI1 localization in 7% and 10% of cells, respectively, for OP-6 II5 and OP-596 II2). In 100% of cells counted for all other mutant individuals, DNAI1 is not detected **(B)** Table describing the total number of cells counted per group and localization pattern observed. The quantification analysis for respiratory cilia represents at least two independent experiments.
