# Supplementary material for: Defects in the cytoplasmic assembly of axonemal dynein arms cause morphological abnormalities and dysmotility in sperm cells leading to male infertility
Source: PLoS Genet. 2021 Feb 26;17(2):e1009306. doi: 10.1371/journal.pgen.1009306 (PMC7909641; doi:10.1371/journal.pgen.1009306)
Supplement: S9 Fig — (PDF) [file pgen.1009306.s009.pdf]

A

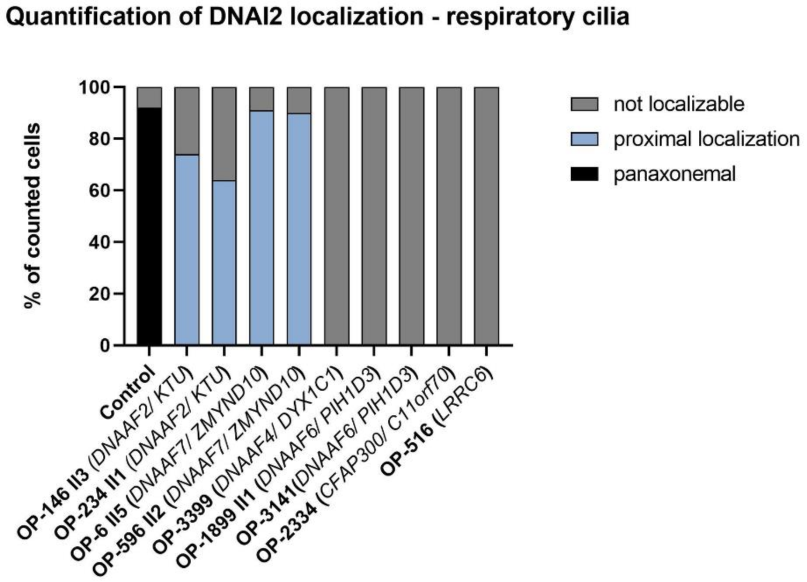

B

	panaxonemal	proximal localization	not localizable	n° counted cells	replicates
**Control**	322	0	27	349	3
**OP-146 II3 (*DNAAF2/ KTU*)**	0	28	10	38	2
**OP-234 II1 (*DNAAF2/ KTU*)**	0	130	74	204	3
**OP-6 II5 (*DNAAF7/ ZMYND10*)**	0	193	20	213	3
**OP-596 II2 (*DNAAF7/ ZMYND10*)**	0	136	15	151	3
**OP-3399 (*DNAAF4/ DYX1C1*)**	0	0	205	205	3
**OP-1899 II1 (*DNAAF6/ PIH1D3*)**	0	0	233	233	3
**OP-3141 (*DNAAF6/ PIH1D3*)**	0	0	21	21	3
**OP-2334 (*CFAP300/ C11orf70*)**	0	0	193	193	3
**OP-516 (*LRRC6*)**	0	0	122	122	3

S9 Fig. Quantification of DNAI2 immunofluorescence (IF) in respiratory cells of healthy control and dynein preassembly mutant individuals. (A) Graph representing the quantification analysis performed for the DNAI2 IF on respiratory cilia. In control nasal brush biopsies, DNAI2 is localized along the entire ciliary length in 92% of counted cells, whereas in 8% of cells immunoreactivity against DNAI2 is not detected. In *DNAAF2*-mutant individuals OP-146II3 and OP-243II1, a proximal localization of DNAI2 is observed in 74% and 64% of counted cells, respectively, whereas the remaining cells counted (26% for OP-146 II3 and 36% for OP-234 II1) do not display immunoreactivity against DNAI2. In addition, *DNAAF7*-mutant individuals OP-6 II5 and OP-596 II2 show a proximal localization pattern for DNAI2 in 91% and 90% of their respiratory cilia (no DNAI2 localization in 9% and 10% of cells, respectively, for OP-6 II5 and OP-596 II2). In 100% of cells counted for all other mutant individuals, DNAI2 is not detected **(B)** Table describing the total number of cells counted per group and localization pattern observed. The quantification analysis for respiratory cilia represents at least two independent experiments.
