# Supplementary material for: Defects in the cytoplasmic assembly of axonemal dynein arms cause morphological abnormalities and dysmotility in sperm cells leading to male infertility
Source: PLoS Genet. 2021 Feb 26;17(2):e1009306. doi: 10.1371/journal.pgen.1009306 (PMC7909641; doi:10.1371/journal.pgen.1009306)
Supplement: S10 Fig — (PDF) [file pgen.1009306.s010.pdf]

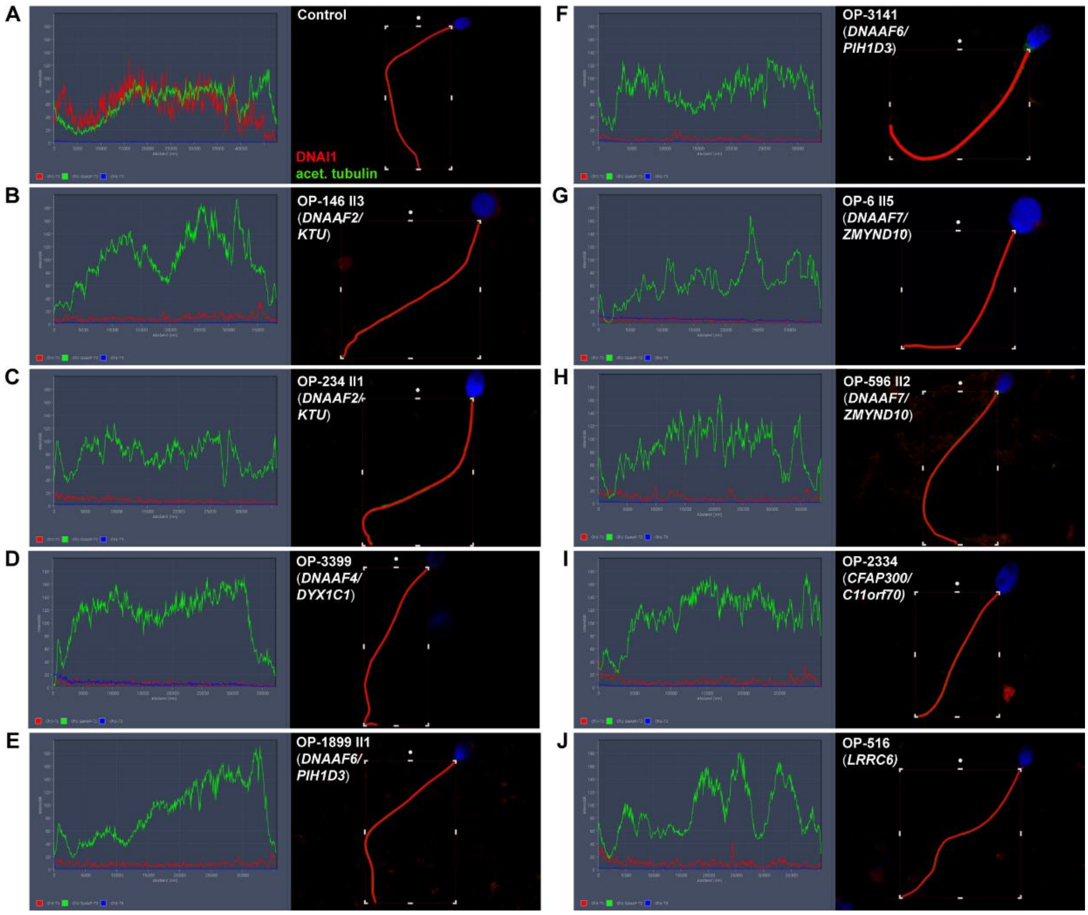

S10 Fig. Measurement of the DNAI1 fluorescence intensity along the flagellar axonemes of control and dynein preassembly mutant sperm. (A) Intensity profile of DNAI1 signal (red) shows a normal distribution of DNAI1 along the flagellar axoneme in control sperm. The intensity profile is co-localizing with the intensity profile of the green signal (acetylated tubulin, used as flagellar marker). **(B-J)** In *DNAAF2/KTU* (OP-146 II3, OP-234 II1)-, *DNAAF4/DYX1C1* (OP-3399)-, *DNAAF6/PIH1D3* (OP-1899 II1, OP-3141)-, *DNAAF7/ZMYND10* (OP-6 II5, OP-596 II2)-, *CFAP300/C11orf70* (OP-2334)- and *LRRC6* (OP-516)-mutant individuals, the intensity profile of DNAI1 signal (red) shows absence or severe reduction of DNAI1 in flagellar axonemes, when compared to control sperm**.** The red line along the sperm flagella (within the dashed box) indicate the path of the intensity profile. One representative example is shown per individual.
