# Supplementary material for: Defects in the cytoplasmic assembly of axonemal dynein arms cause morphological abnormalities and dysmotility in sperm cells leading to male infertility
Source: PLoS Genet. 2021 Feb 26;17(2):e1009306. doi: 10.1371/journal.pgen.1009306 (PMC7909641; doi:10.1371/journal.pgen.1009306)
Supplement: S12 Fig — (PDF) [file pgen.1009306.s012.pdf]

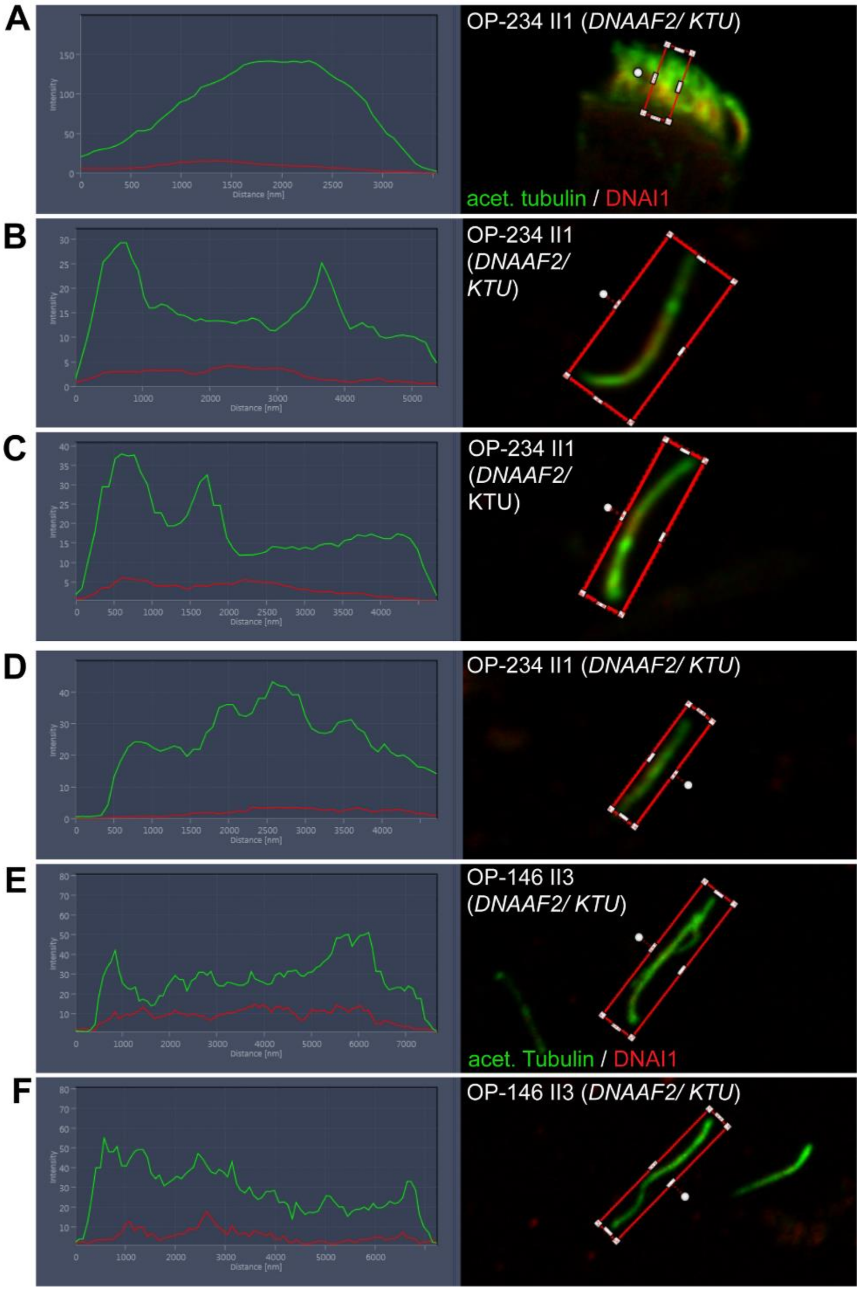


**S12 Fig. Measurement of the DNAI1 fluorescence intensity along the ciliary axonemes of *DNAAF2/KTU*-mutant respiratory cells.** Intensity profile of DNAI1 signal (red) shows in *DNAAF2/KTU*-mutant individuals OP-146 II3 and OP-234 II1 a variable signal distribution along the ciliary axoneme. The signal results to be either reduced in the distal ciliary compartment compared to the proximal part or severely reduced along the entire ciliary axoneme. In the first case, the proximal localized signal results to be reduced of about 60-90% compared to control samples (S11 Fig). The red boxes indicate the path of the intensity profile. Six representative examples are shown.
