# Supplementary material for: Defects in the cytoplasmic assembly of axonemal dynein arms cause morphological abnormalities and dysmotility in sperm cells leading to male infertility
Source: PLoS Genet. 2021 Feb 26;17(2):e1009306. doi: 10.1371/journal.pgen.1009306 (PMC7909641; doi:10.1371/journal.pgen.1009306)
Supplement: S13 Fig — (PDF) [file pgen.1009306.s013.pdf]

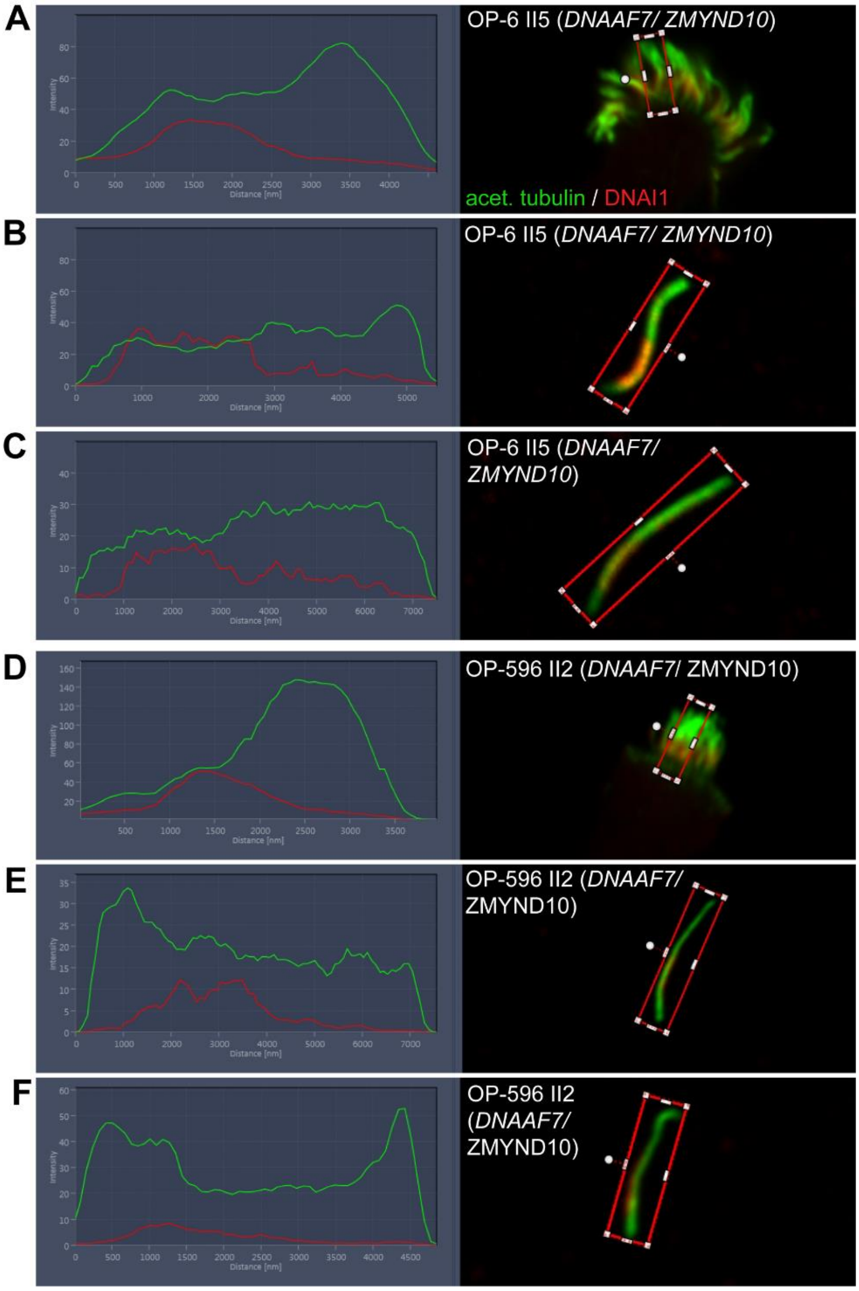

S13 Fig. Measurement of the DNAI1 fluorescence intensity along the ciliary axonemes of *DNAAF7/ZMYND10*-mutant respiratory cells. Intensity profile of DNAI1 signal (red) shows a reduction in the distal part of ciliary axoneme in *DNAAF7/ZMYND10*-mutant cilia (OP-6 II5 and OP-596 II2). The DNAI1 signal in the proximal ciliary length results either comparable to control samples or reduced up to 60-80% (S11 Fig). The red boxes indicate the path of the intensity profile. Six representative examples are shown.
