# Supplementary material for: Defects in the cytoplasmic assembly of axonemal dynein arms cause morphological abnormalities and dysmotility in sperm cells leading to male infertility
Source: PLoS Genet. 2021 Feb 26;17(2):e1009306. doi: 10.1371/journal.pgen.1009306 (PMC7909641; doi:10.1371/journal.pgen.1009306)
Supplement: S15 Fig — (PDF) [file pgen.1009306.s015.pdf]

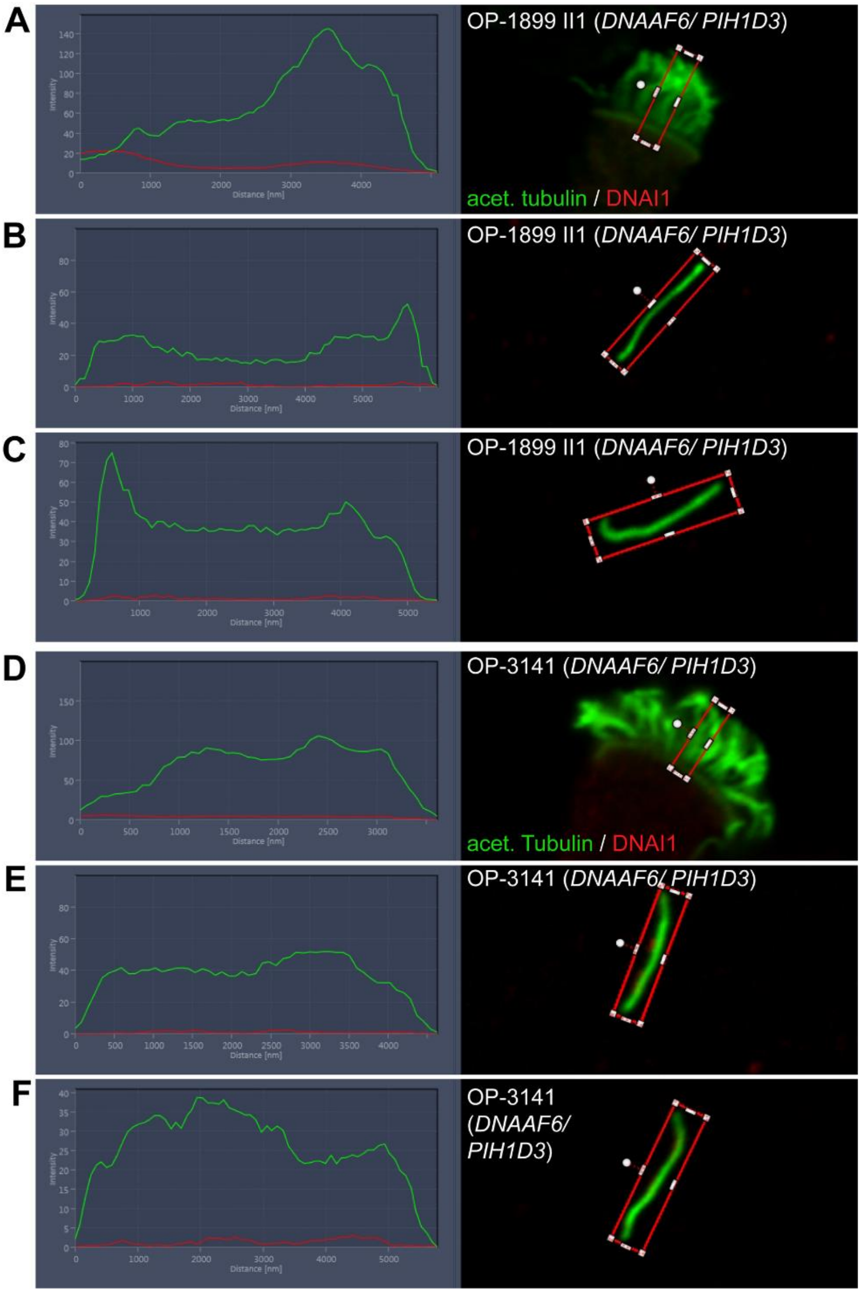

S15 Fig. Measurement of the DNAI1 fluorescence intensity along the ciliary axonemes of *DNAAF6/PIH1D3*-mutant respiratory cells. Intensity profile of DNAI1 signal (red) shows absence or severe reduction of DNAI1 in ciliary axonemes of *DNAAF6/PIH1D3*-mutant cilia (OP-1899 II1 and OP-3141) when compared to control cells (S11 Fig). The red boxes indicate the path of the intensity profile. Six representative examples are shown.
