# Supplementary material for: Defects in the cytoplasmic assembly of axonemal dynein arms cause morphological abnormalities and dysmotility in sperm cells leading to male infertility
Source: PLoS Genet. 2021 Feb 26;17(2):e1009306. doi: 10.1371/journal.pgen.1009306 (PMC7909641; doi:10.1371/journal.pgen.1009306)
Supplement: S16 Fig — (PDF) [file pgen.1009306.s016.pdf]

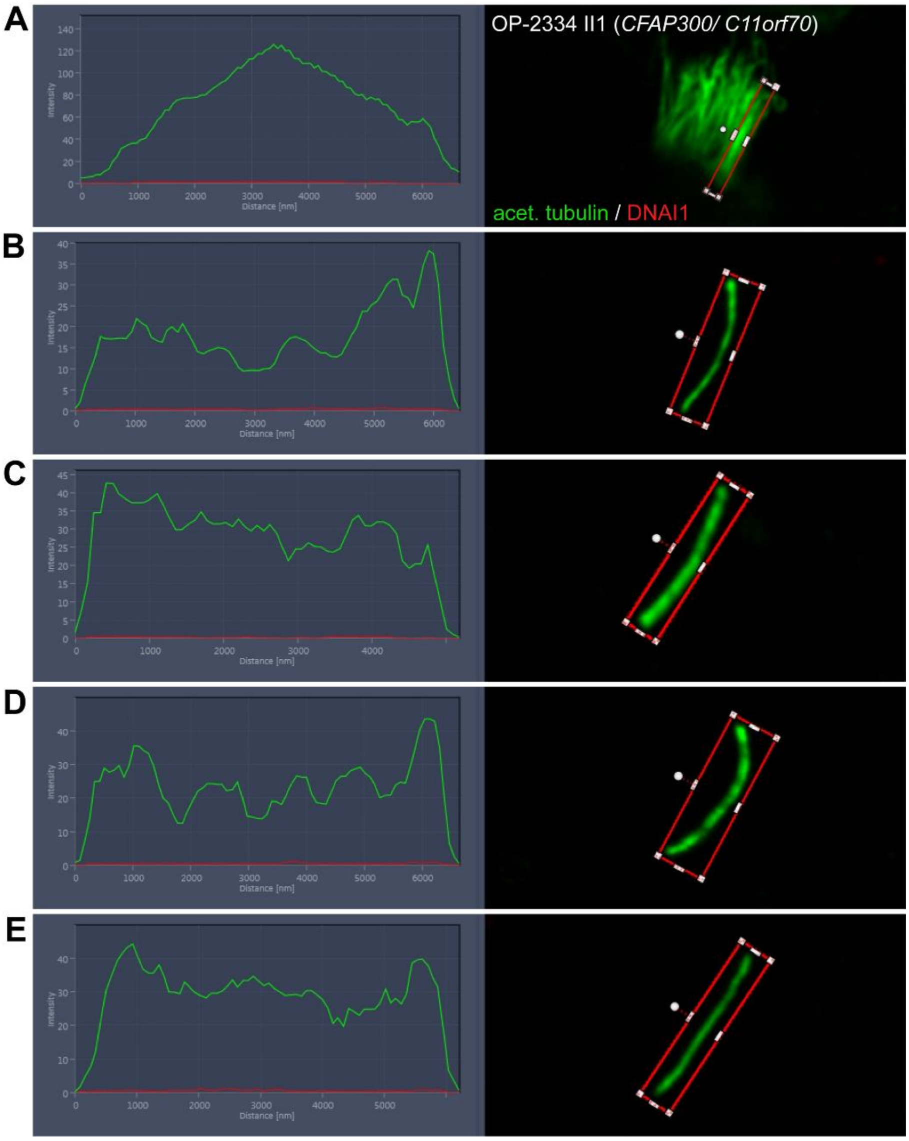


**S16 Fig. Measurement of the DNAI1 fluorescence intensity along the ciliary axonemes of *CFAP300/C11orf70*-mutant respiratory cells.** Intensity profile of DNAI1 signal (red) shows absence of DNAI1 in ciliary axonemes of *CFAP300/C11orf70*-mutant cilia (OP-2334) when compared to control cells (S11 Fig). The red boxes indicate the path of the intensity profile. Five representative examples are shown.
