# Supplementary material for: Defects in the cytoplasmic assembly of axonemal dynein arms cause morphological abnormalities and dysmotility in sperm cells leading to male infertility
Source: PLoS Genet. 2021 Feb 26;17(2):e1009306. doi: 10.1371/journal.pgen.1009306 (PMC7909641; doi:10.1371/journal.pgen.1009306)
Supplement: S17 Fig — (PDF) [file pgen.1009306.s017.pdf]

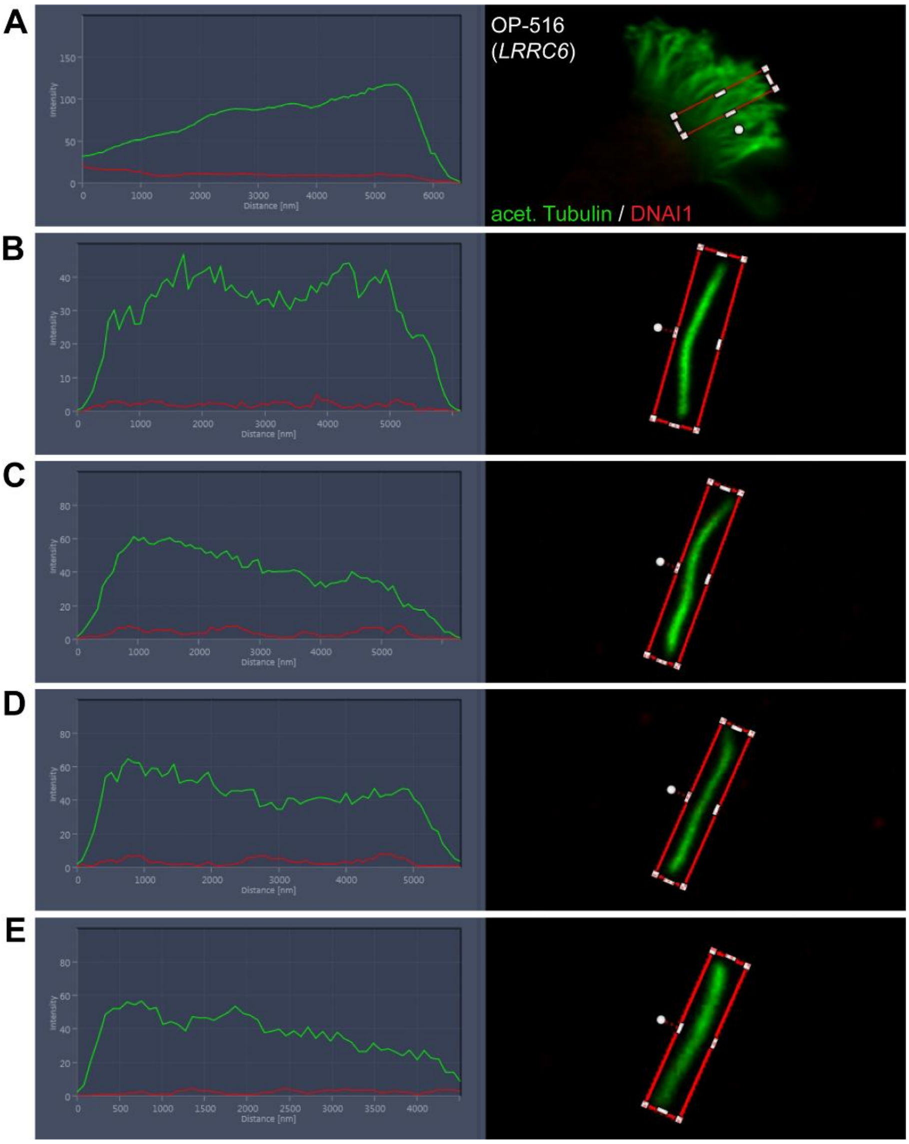

S17 Fig. Measurement of the DNAI1 fluorescence intensity along the ciliary axonemes of *LRRC6*-mutant respiratory cells. Intensity profile of DNAI1 signal (red) shows absence or severe reduction of DNAI1 in ciliary axonemes of *LRRC6*-mutant cilia (OP-516), when compared to control cells (S11 Fig). The red boxes indicate the path of the intensity profile. Five representative examples are shown.
