# Supplementary material for: Defects in the cytoplasmic assembly of axonemal dynein arms cause morphological abnormalities and dysmotility in sperm cells leading to male infertility
Source: PLoS Genet. 2021 Feb 26;17(2):e1009306. doi: 10.1371/journal.pgen.1009306 (PMC7909641; doi:10.1371/journal.pgen.1009306)
Supplement: S19 Fig — (PDF) [file pgen.1009306.s019.pdf]

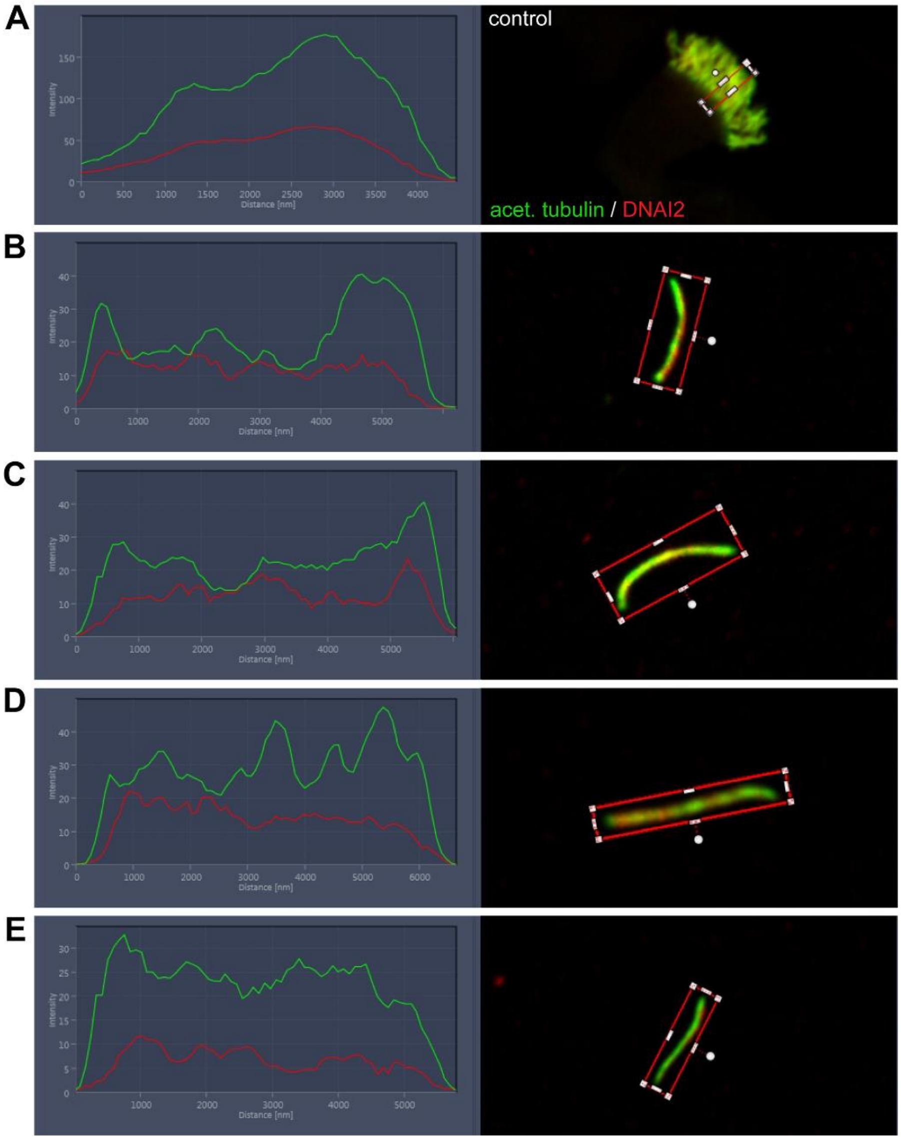


**S19 Fig. Measurement of the DNAI2 fluorescence intensity along the ciliary axonemes of control respiratory cells.** Intensity profile of DNAI2 signal (red) shows a normal distribution of DNAI2 along the ciliary axoneme in control samples of ciliated respiratory cells and single cilia. The red boxes indicate the path of the intensity profile. Five representative examples are shown.
