# Supplementary material for: Defects in the cytoplasmic assembly of axonemal dynein arms cause morphological abnormalities and dysmotility in sperm cells leading to male infertility
Source: PLoS Genet. 2021 Feb 26;17(2):e1009306. doi: 10.1371/journal.pgen.1009306 (PMC7909641; doi:10.1371/journal.pgen.1009306)
Supplement: S20 Fig — (PDF) [file pgen.1009306.s020.pdf]

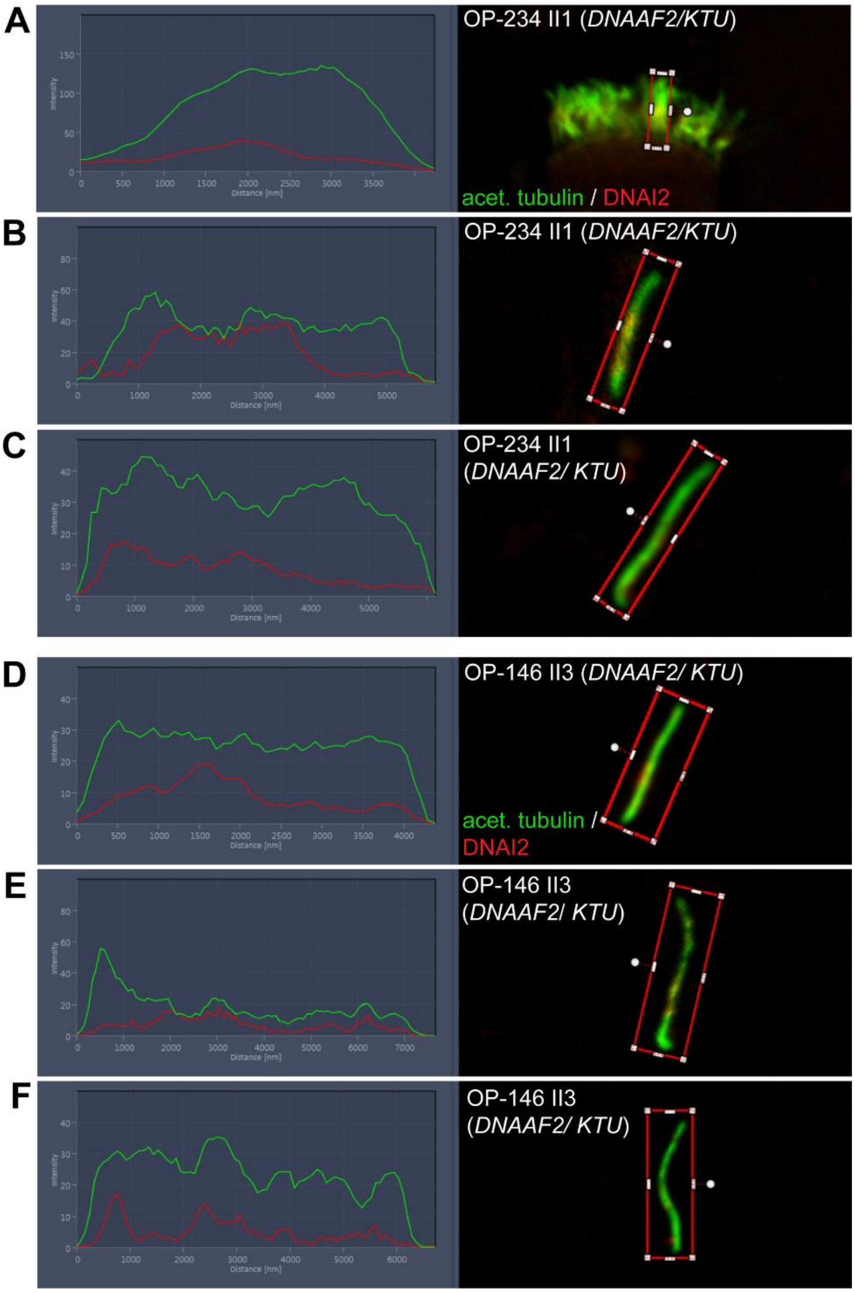

S20 Fig. Measurement of the DNAI2 fluorescence intensity along the ciliary axonemes of *DNAAF2/KTU*-mutant respiratory cells. Intensity profile of DNAI2 signal (red) shows a reduction in the distal part of ciliary axonemes in *DNAAF2/KTU*-mutant cilia (OP-146 II3 and OP-234 II1), when compared to control cells (S19 Fig). Alternatively, a severe reduction of DNAI1 is observed along the entire ciliary axoneme, with single intensity peaks comparable to the intensity in control cells. The red boxes indicate the path of the intensity profile. Six representative examples are shown.
