# Supplementary material for: Defects in the cytoplasmic assembly of axonemal dynein arms cause morphological abnormalities and dysmotility in sperm cells leading to male infertility
Source: PLoS Genet. 2021 Feb 26;17(2):e1009306. doi: 10.1371/journal.pgen.1009306 (PMC7909641; doi:10.1371/journal.pgen.1009306)
Supplement: S21 Fig — (PDF) [file pgen.1009306.s021.pdf]

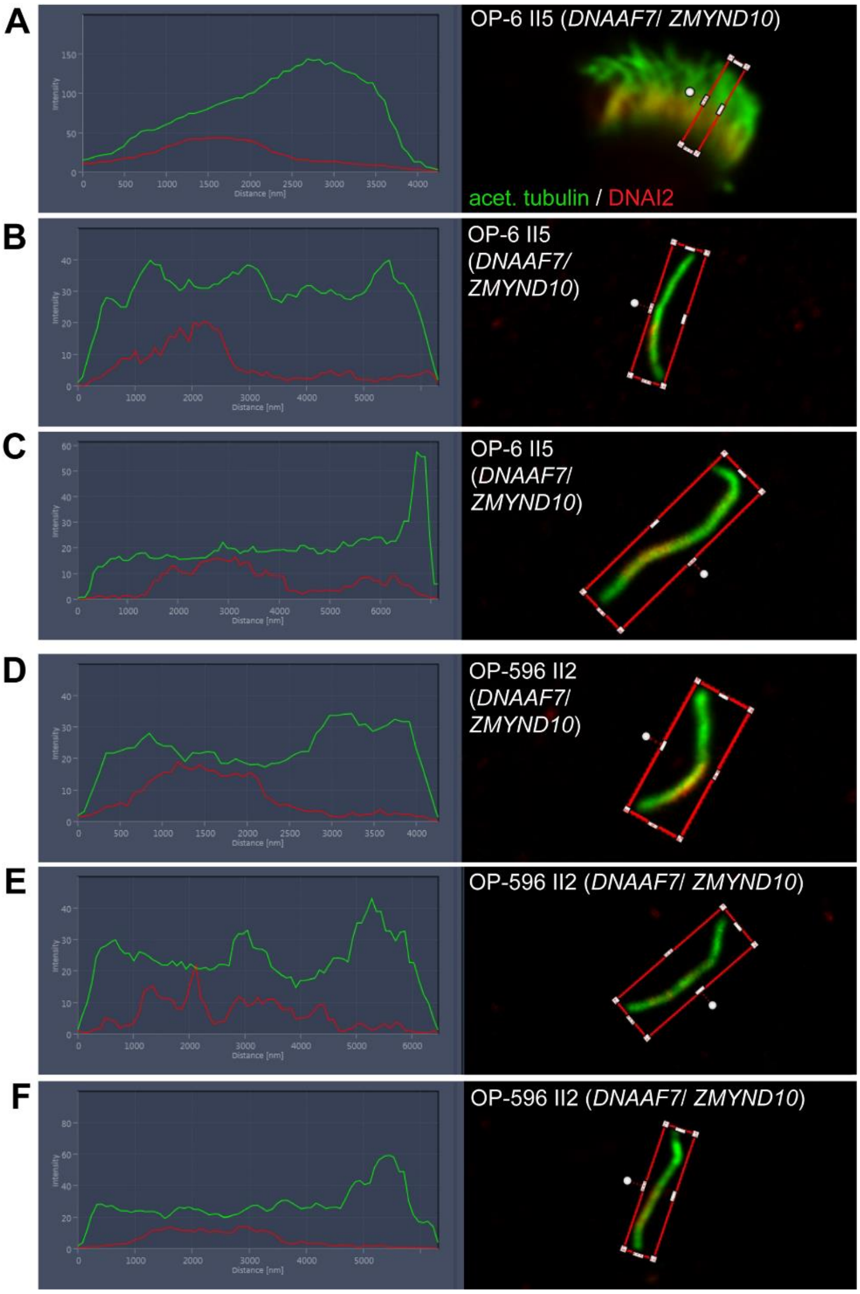

S21 Fig. Measurement of the DNAI2 fluorescence intensity along the ciliary axonemes of *DNAAF7/ZMYND10*-mutant respiratory cells. Intensity profile of DNAI2 signal (red) shows a reduction in the distal part of ciliary axoneme in *DNAAF7/ZMYND10*-mutant cilia (OP-6 II5 and OP-596 II2). The DNAI2 signal in the proximal ciliary length results comparable to control samples (S19 Fig). The red boxes indicate the path of the intensity profile. Five representative examples are shown.
