# Supplementary material for: Defects in the cytoplasmic assembly of axonemal dynein arms cause morphological abnormalities and dysmotility in sperm cells leading to male infertility
Source: PLoS Genet. 2021 Feb 26;17(2):e1009306. doi: 10.1371/journal.pgen.1009306 (PMC7909641; doi:10.1371/journal.pgen.1009306)
Supplement: S26 Fig — (PDF) [file pgen.1009306.s026.pdf]

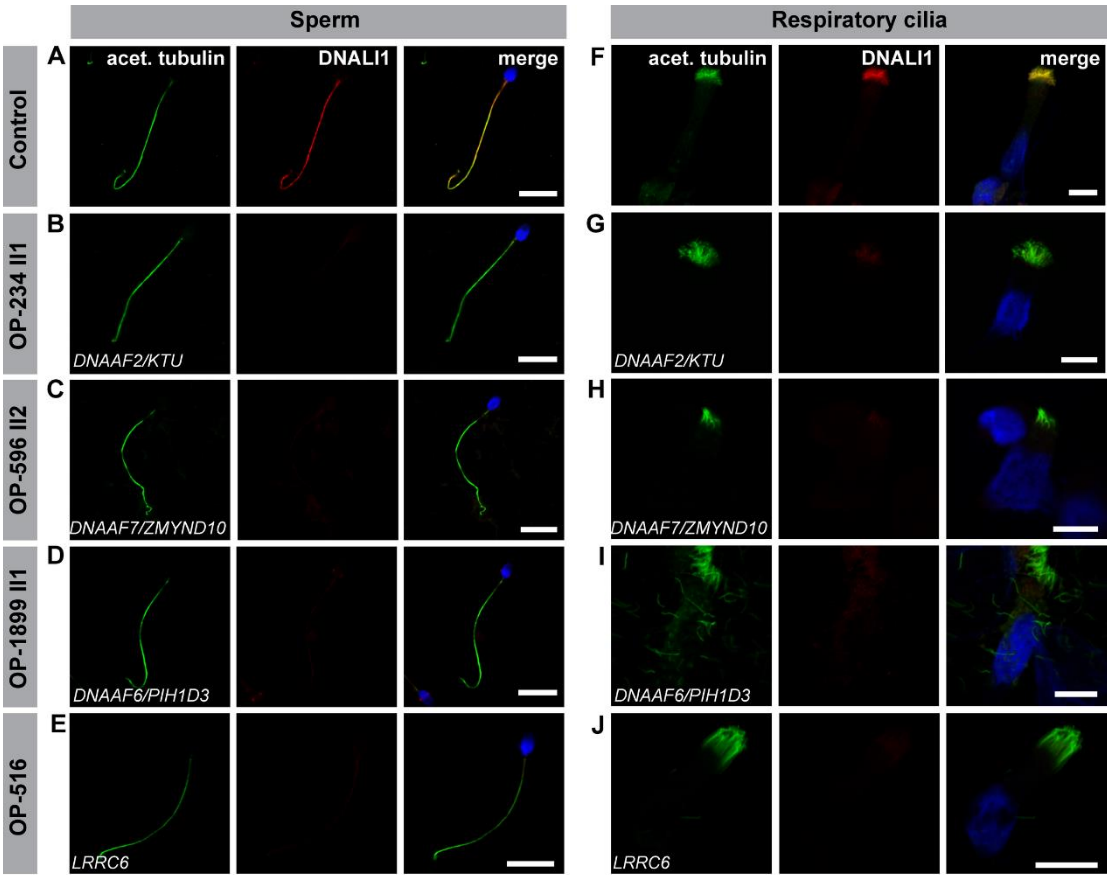

S26 Fig. Mutant sperm flagella and respiratory cilia show absence or reduction of the IDA intermediate chain DNALI1. Control **(A)** and mutant **(B-E)** sperm cells, as well as control **(F)** and mutant **(G-J)** respiratory cells were double-labeled with antibodies directed against acetylated α tubulin (green) and the inner dynein arm intermediate light chain DNALI1 (red). Both antibodies co-localize along the flagella and cilia in cells from the unaffected control (yellow, **A**, **F**). In all mutant sperm and respiratory cells DNALI1 was not detected along the flagellar and ciliary axoneme (**B**-**E**, and **G**-**J**). Nuclei were stained with Hoechst33342 (blue). Scale bars represent 10 µm.
