# Supplementary material for: Defects in the cytoplasmic assembly of axonemal dynein arms cause morphological abnormalities and dysmotility in sperm cells leading to male infertility
Source: PLoS Genet. 2021 Feb 26;17(2):e1009306. doi: 10.1371/journal.pgen.1009306 (PMC7909641; doi:10.1371/journal.pgen.1009306)
Supplement: S1 Table — (PDF) [file pgen.1009306.s027.pdf]

S1 Table. Primer pairs used for amplification of *DNAAF6* exons and DNAI1 exon 3 in OP-3141.

Fw, forward; Rv, reverse; bp, base pair; ic, internal control.

**Name**	**Sequence 5' – 3'**	**Size**
*DNAAF6*_Exon 1Fw	TTGGCCAGAAAAGTCAAGCG	283 bp
DNAAF6 _Exon 1Rv	TTCTCCACCTTGCCCAACTT	
DNAAF6 _Exon2 Fw	CCAATGGAGTGATCCAGGGAA	399 bp
DNAAF6 _Exon2 Rv	TCAGCCTTGTAGCTTCCTGTG	
DNAAF6 _Exon3 Fw	CACTGAGCTGGAACTAGGGT	406 bp
DNAAF6 _Exon3 Rv	CTCCCTATAATTTCTCTACCAAGGA	
DNAAF6 _Exon4 Fw	GTGTGAAGAAGAACAGGAGCA	317 bp
DNAAF6 _Exon4 Rv	CATGCCTTCCGTGTGTCAC	
DNAAF6 _Exon5 Fw	CTGAAGACCTGCAAGCACTT	362 bp
DNAAF6 _Exon5 Rv	CGTGAAACATAGCAACCACGA	
DNAAF6 _Exon6 Fw	TGGAGCCAGAAACCTTAGTCA	448 bp
DNAAF6 _Exon6 Rv	TTTTCTTCAGACTTTAGGGGTGA	
DNAAF6 _Exon7 Fw	AAATAGGGGCGCCAAAACAA	592 bp
DNAAF6 _Exon7 Rv	TTCGGCCTCCCAAAGTGTT	
DNAI1_ic_Fw	TGAATGAAGGGGCTTTCTGT	266 bp
DNAI1_ic_Rv	TCTGGGCAAGCTCCTAATCA	
