# Supplementary material for: Defects in the cytoplasmic assembly of axonemal dynein arms cause morphological abnormalities and dysmotility in sperm cells leading to male infertility
Source: PLoS Genet. 2021 Feb 26;17(2):e1009306. doi: 10.1371/journal.pgen.1009306 (PMC7909641; doi:10.1371/journal.pgen.1009306)
Supplement: S2 Table — (PDF) [file pgen.1009306.s028.pdf]

S2 Table. Primer sequences used for Sanger Sequencing. Fw, forward; Rv, reverse; bp, base pair.

Name	Sequence 5’ – 3’	Size
DNAAF4 Exon 2 Fw	GGTAACCCCAAGAATCGGCA	549 bp
DNAAF4 Rv	CCAGGGCTTCCTCATATGCC	
DNAAF7 Exon1 Fw	GCAACTGTCCTGTCCCAGAC	455 bp
DNAAF7 Exon1 Rv	GTCCACGTGTGTGCATTAGG	
DNAAF7 Exon5-6 Fw	AGGGAGATTGTGTCTGTGTGG	434 bp
DNAAF7 Exon5-6 Rv	CTTCCAGCCCTCCACAGTAG	
LRRC6 Exon5 Fw	CTTTTGGGAAGTGCCATTGT	967 bp
LRRC6 Exon5 Rv	TTGCATCATAAAACATTCTATTTTCTG	
