# Supplementary material for: Defects in the cytoplasmic assembly of axonemal dynein arms cause morphological abnormalities and dysmotility in sperm cells leading to male infertility
Source: PLoS Genet. 2021 Feb 26;17(2):e1009306. doi: 10.1371/journal.pgen.1009306 (PMC7909641; doi:10.1371/journal.pgen.1009306)
Supplement: S3 Table — (PDF) [file pgen.1009306.s029.pdf]

S3 Table. List of 33 genes in customized PCD panel.

Gene symbol	RefSeq database accession number - transcript
ARMC4	(NM_018076.4)
CFAP298	(NM_021254.2)
CCDC103	(NM_213607.2)
CCDC11	(NM_145020.4)
CCDC114	(NM_144577.3)
CCDC151	(NM_145045.4)
CCDC164	(NM_145038.4)
CCDC39	(NM_181426.1)
CCDC40	(NM_017950.3)
CCDC65	(NM_033124.4)
CCNO	(NM_021147.4)
DNAAF1	(NM_178452.5)
DNAAF2	(NM_018139.2)
DNAAF3	(NM_001256714.1)
DNAAF4	(NM_130810.3)
DNAH11	(NM_001277115.1)
DNAH5	(NM_001369.2)
DNAH9	(NM_001372.3)
DNAI1	(NM_012144.3)
DNAI2	(NM_023036.4)
DNAL1	(NM_031427.3)
GAS8	(NM_001286209.1)
DNAAF5	(NM_017802.3)
HYDIN	(NM_001270974.1)
LRRC6	(NM_012472.5)
MCIDAS	(NM_001190787.1)
NME8	(NM_016616.4)
RPGR	(NM_000328.2)
RSPH1	(NM_080860.3)
RSPH3	(NM_031924.4)
RSPH4A	(NM_001010892.2)
RSPH9	(NM_152732.4)
SPAG1	(NM_172218.2)
